# Protein-primed homopolymer synthesis by an antiviral reverse transcriptase

**DOI:** 10.1038/s41586-025-09179-5

**Published:** 2025-05-28

**Authors:** Stephen Tang, Rimantė Žedaveinytė, Nathaniel Burman, Shishir Pandey, Josephine L. Ramirez, Louie M. Kulber, Tanner Wiegand, Royce A. Wilkinson, Yanzhe Ma, Dennis J. Zhang, George D. Lampe, Mirela Berisa, Marko Jovanovic, Blake Wiedenheft, Samuel H. Sternberg

**Affiliations:** 1 Department of Biochemistry and Molecular Biophysics, Columbia University, New York, NY, USA.; 2 Department of Microbiology and Cell Biology, Montana State University, Bozeman, MT, USA.; 3 Howard Hughes Medical Institute, Columbia University, New York, NY, USA.; 4 Department of Biological Sciences, Columbia University, New York, NY, USA.; 5 Metabolomics Core, Icahn School of Medicine at Mount Sinai, New York, NY, USA.

## Abstract

Bacteria defend themselves from viral predation using diverse immune systems, many of which target foreign DNA for degradation^1^. Defense-associated reverse transcriptase (DRT) systems provide an intriguing counterpoint to this strategy by using DNA synthesis instead^2,3^. We and others recently showed that DRT2 systems use an RNA template to assemble a *de novo* gene that encodes the antiviral effector protein Neo^4,5^. It remains unclear whether similar mechanisms of defense are used by other related DRT families. Here, we show that DRT9 systems defend against phage using DNA homopolymer synthesis. Viral infection triggers polydeoxyadenylate (poly-dA) accumulation in the cell, driving abortive infection and population-level immunity. Cryo-EM structures reveal how a noncoding RNA serves as both a structural scaffold and reverse transcription template to direct hexameric complex assembly and poly-dA synthesis. Notably, biochemical and functional experiments identify tyrosine residues within the reverse transcriptase itself that probably prime DNA synthesis, leading to the formation of protein–DNA covalent adducts. Synthesis of poly-dA by DRT9 *in vivo* is regulated by the competing activities of phage-encoded triggers and host-encoded silencers. Collectively, our study identifies a nucleic-acid-driven defense system that expands the paradigm of bacterial immunity and broadens the known functions of reverse transcriptases.

Bacteria encode diverse mechanisms for antiviral defense, the majority of which degrade invading nucleic acids using either programmable, sequence-specific, or non-specific nucleases^1^. Also common are enzymes that act on host nucleic acids — synthesizing second messenger molecules^6^, converting nucleotides into suicide/terminator analogs^7^, or depleting nucleotides altogether^8,9^. Against this backdrop, reverse transcriptase (RT) enzymes, which polymerize rather than degrade DNA, would seem unlikely contributors to antiphage defense. And yet, multiple families of RTs have recently been implicated in defensive functions spanning the adaptive and innate arms of the bacterial immune system^2–5,10,11^.

RTs associated with CRISPR-Cas comprise one family of defense-related systems, leveraging RNA-templated DNA synthesis to facilitate spacer acquisition directly from viral RNA transcripts^10^. But whereas CRISPR-associated RTs reverse transcribe foreign RNA, other RT systems encode and reverse transcribe their own non-coding RNA (ncRNA). Retron-encoded ncRNAs template the synthesis of hybrid RNA–DNA molecules — known as multicopy single-stranded DNA (msDNA) — that serve as antitoxins against host-encoded toxins^12^. Upon viral infection, a cascade of events leads to msDNA modification or degradation and toxin release, killing the infected cell and thereby preempting further viral propagation^12,13^. This strategy, known as abortive infection, protects the larger bacterial population at the cost of the infected cell, and is ubiquitous among innate immune mechanisms^1^. Abortive infection also characterizes a third group of antiviral RTs typified by AbiK, AbiA, and AbiP2, which, unlike CRISPR-associated RTs and retrons, have been shown to synthesize random DNA polymers independently of any RNA template^14,15^. Intriguingly, recent structural and biochemical studies revealed that DNA polymerization by Abi RTs is primed by a tyrosine residue within the RT itself and requires the formation of a homo-oligomeric RT complex^15,16^. These studies have demonstrated the presence of highly unusual biochemical behaviors across the Abi RT family, but how these activities relate to phage infection, in terms of their regulation and their effects on the phage and/or the host, remains unresolved.

The Abi RTs belong to a large and diverse family of systems collectively designated as the Unknown Group (UG)^17^. Pioneering work by Gao et al. and Mestre et al. revealed an expanded set of UG systems that function in antiviral immunity, termed defense-associated RTs (DRT)^2,3^. We and others recently resolved the molecular mechanism of the DRT2 defense system^4,5^. Like retron systems, DRT2 operons encode a conserved, structured ncRNA, which provides the template for complementary DNA (cDNA) synthesis by the RT. Rolling circle reverse transcription of the ncRNA leads to the synthesis of concatemeric cDNA (ccDNA) products, assembling intact promoters and a nearly endless open reading frame (*neo*) spanning ccDNA repeats. During phage infection, ccDNA is transcribed and translated to yield Neo proteins that drive cell growth arrest in a mechanism akin to abortive infection. Altogether, these studies have provided an early glimpse into the remarkable and unique molecular functions of UG-family RTs, although it is unresolved whether similar mechanisms exist across the vast diversity of unexplored UG systems.

We set out to uncover conserved mechanisms of RNA-templated *de novo* gene assembly in other bacterial immune systems. In this regard, we focused on DRT9, because of its nearby placement on the phylogenetic tree of UG RTs and its similar genetic architecture to DRT2 (Fig. 1a). What we instead found was a fundamentally distinct mechanism of RT-mediated innate immunity. Here we show that DRT9 systems are triggered by phage infection to produce long, uninterrupted chains of polydeoxyadenylate (poly-dA). Poly-dA synthesis is primed by tyrosine residues within the RT itself, and is templated by a structured ncRNA containing a universally conserved poly-uridine tract. Cryo-electron microscopy (cryo-EM) data reveal extensive interactions between protein and RNA components that mediate the assembly of a hexameric RT-ncRNA complex, in which the templating uridines reside proximal to the RT active site. The production of poly-dA is sensitively regulated by a ‘tug-of-war’ between host- and phage-encoded factors, and its accumulation in cells drives cell death and abortive infection. In addition to revealing an elegant and intricate mechanism by which DNA synthesis confers antiviral immunity, our work highlights an unusual synthetic pathway and biological function of a nucleic acid homopolymer.

## DRT9 synthesizes DNA homopolymers

**Extended Data Figure 1 |:**
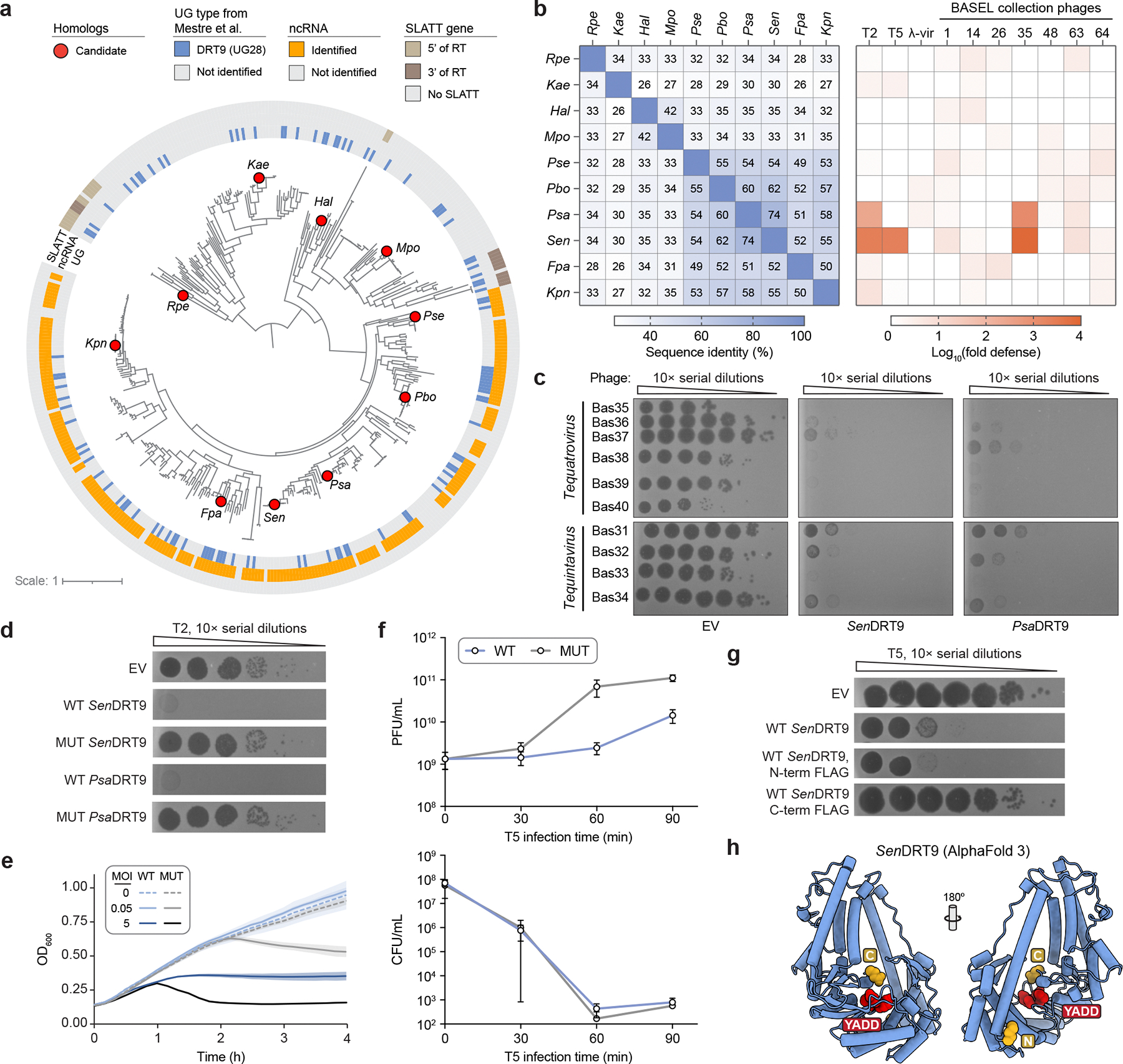
Screening and identification of active DRT9 immune systems. **a,** Phylogenetic tree of DRT9-encoded RT homologs. Outer rings show SLATT protein and ncRNA association within nearby genomic neighborhoods, and the inner ring shows previously identified DRT9 (UG28) homologs^3^; systems selected for experimental testing are indicated with red circles. **b,** Heatmap of pairwise amino acid sequence identity percentages among DRT9-encoded RT homologs tested in this study (left), and heatmap of phage defense activity for the same DRT9 systems tested against 10 diverse *E. coli* phages (right); RT proteins encoded N-terminal FLAG tags. **c,** Representative plaque assays demonstrating that *Sen*DRT9 and *Psa*DRT9 exhibit broad defense against phages from the *Tequatrovirus* and *Tequintavirus* genera, as compared to an empty vector (EV) control. **d,** Plaque assays demonstrating that defense activity against T2 phage is completely abolished for both *Sen*DRT9 and *Psa*DRT9 systems encoding catalytically inactive RT mutants (MUT). **e,** Growth curves of cells expressing WT or MUT *Sen*DRT9 +/− T5 phage at the indicated multiplicity of infection (MOI). Data are shown as mean ± s.d. for n = 3 independent biological replicates. **f,** Plaque forming unit (PFU; top) and colony forming unit (CFU; bottom) measurements from an infection time course experiment in which cells expressing WT or MUT *Sen*DRT9 were infected with T5 phage at a high MOI. Cells expressing the WT defense system attenuated phage replication but were unable to recover from infection, indicating that defense activation leads to abortive infection and cell death. Data are shown as mean ± s.d. for n = 3 independent biological replicates. **g**, Plaque assays demonstrating that *Sen*DRT9 encoding an RT with an N-terminal FLAG, but not C-terminal FLAG, retains WT defense against T5 phage. **h,** AlphaFold 3 structure prediction of the RT monomer from *Sen*DRT9, highlighting the predicted positions of the N- and C-termini (orange spheres) and YADD active site (red spheres).

DRT9 systems encode a reverse transcriptase (RT) gene and an upstream ncRNA, occasionally in association with a SLATT domain-containing protein^3^ (Extended Data Fig. 1a, Supplementary Table 1). We selected 10 diverse systems for gene synthesis, expressed them in *E. coli*, and assessed their antiviral activity against a panel of well-characterized *E. coli* phages (Extended Data Fig. 1b, Supplementary Table 2). DRT9 systems from *Salmonella enterica* and *Photobacterium sanctipauli* (hereafter *Sen*DRT9 and *Psa*DRT9) defended against multiple phages, particularly those from the *Tequatrovirus* and *Tequintavirus* genera, and this protection was abolished by mutation of catalytic aspartate residues in the YADD active site (Extended Data Fig. 1c,d). Next, to determine whether DRT9 provides defense through direct inhibition of phage replication or by abortive infection, we performed phage infection assays in liquid culture. When cultures of *Sen*DRT9-expressing cells were infected with phage T5 at a low multiplicity of infection (MOI), they grew similarly to uninfected cultures, but a high MOI of T5 inhibited cell growth (Extended Data Fig. 1e). Quantification of viable phage particles and cells from high-MOI infections revealed that infected cells expressing DRT9 successfully inhibited phage replication, but were unable to recover from infection, indicating that DRT9 activation leads to programmed cell death and abortive infection (Extended Data Fig. 1f).

**Figure 1 |:**
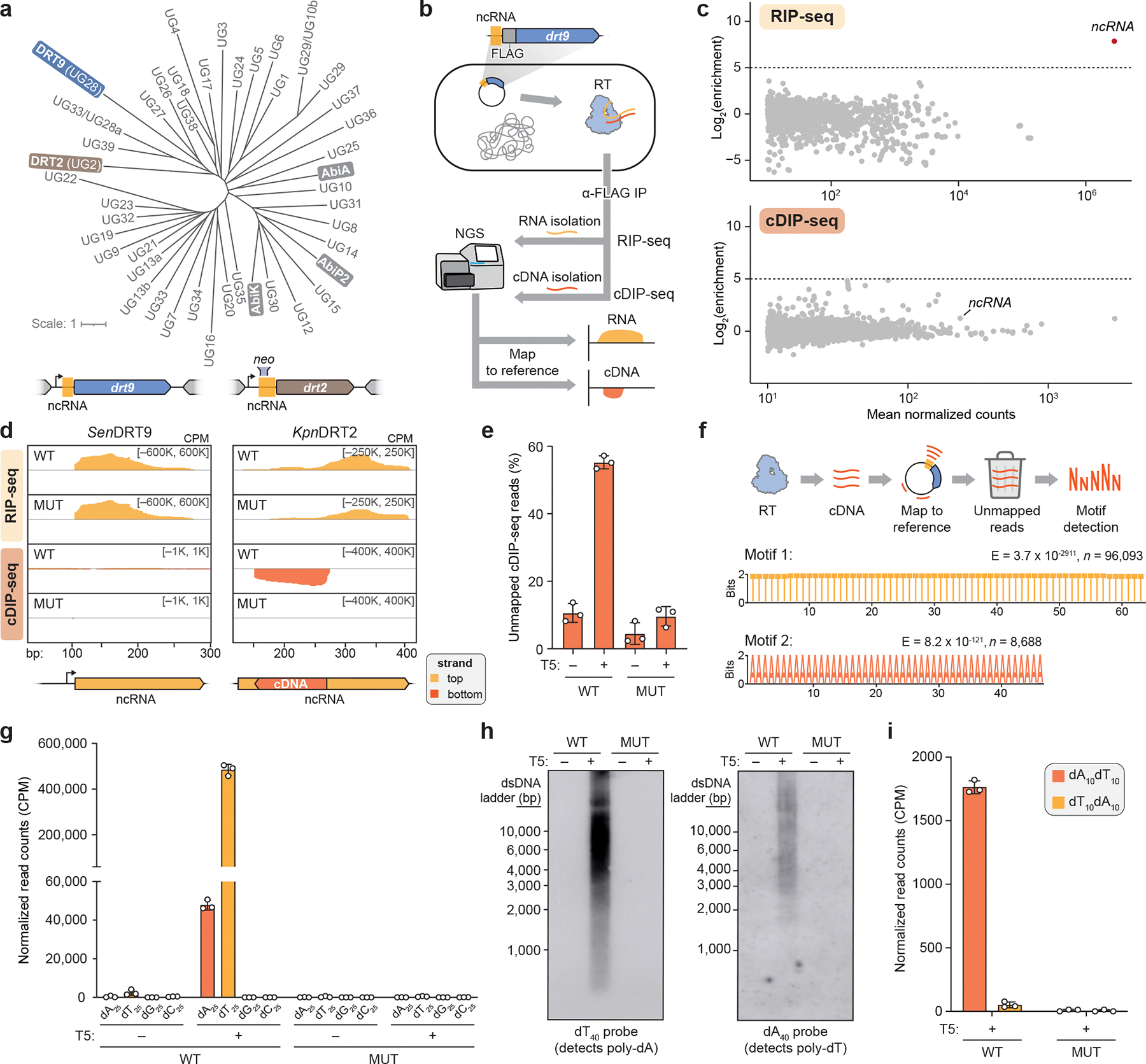
Systematic discovery of DRT9 reverse transcription substrates and products *in vivo*. **a,** Phylogenetic tree of bacterial reverse transcriptase (RT) homologs within the UG family, based on Mestre *et al*.^3^. DRT9 (UG28) and DRT2 (UG2) are highlighted, as are other experimentally studied systems; genetic architectures of archetypal systems are shown below. **b,** Schematic of RNA immunoprecipitation (RIP) and cDNA immunoprecipitation (cDIP) sequencing approaches to identify nucleic acid templates and products of *Sen*DRT9. **c,** MA plots showing enriched RNA (top) and DNA (bottom) loci from RIP-seq and cDIP-seq experiments, respectively, relative to input controls; red dots indicate transcripts with log_2_(enrichment) > 5 and false discovery rate (FDR) < 0.05. **d,** RIP-seq and cDIP-seq coverage tracks for WT or catalytically inactive (MUT) RTs in T5 phage-infected cells expressing *Sen*DRT9 (left) or *Kpn*DRT2 (right). Genomic locus schematics are shown below; data are normalized for sequencing depth and plotted as counts per million reads (CPM). **e,** Bar graph analyzing the percentage of unmapped cDIP-seq reads from WT or MUT *Sen*DRT9 cDIP-seq datasets +/− T5 phage infection. **f,** Schematic of unmapped read analytical pipeline (top), and MEME results that revealed poly-dT and poly-dA motifs enriched in unmapped reads from the WT + T5 cDIP-seq dataset in **e** (bottom). *E*, E-value significance; *n*, number of contributing sites. **g,** Bar graph of normalized homopolymer counts from WT and MUT cDIP-seq datasets +/− T5 phage infection. **h,** Southern blot analysis of total DNA isolated from cells expressing WT or MUT *Sen*DRT9 +/− T5 phage infection. Duplicate blots were probed with either oligo-dT_40_ (left) or oligo-dA_40_ (right); sizes from a double-stranded DNA (dsDNA) ladder are marked. **i,** Bar graph of chimeric dA_10_dT_10_ and dT_10_dA_10_ counts from WT and MUT cDIP-seq datasets in the presence of T5 phage infection. For **e-g,i**, data are mean ± s.d. for n = 3 independent biological replicates.

We next sought to investigate DRT9 reverse transcriptase activity *in vivo* using a FLAG-tagged *Sen*DRT9 RT (*Sen*RT). Interestingly, epitope tagging at the *Sen*RT C-terminus, but not the N-terminus, severely impaired phage defense (Extended Data Fig. 1g). This observation was consistent with AlphaFold 3 modelling of *Sen*RT, which positioned the C-terminus proximal to the predicted catalytic residues, suggesting a potential role for the C-terminus in *Sen*RT enzymatic function (Extended Data Fig. 1h). Next, having confirmed that N-terminally FLAG-tagged *Sen*RT retains wild-type (WT) defense function, we leveraged the tagged RT for RNA immunoprecipitation (RIP) and cDNA immunoprecipitation (cDIP) sequencing^4^ in uninfected and T5 phage-infected cells (Fig. 1b). As with DRT2, the ncRNA encoded immediately upstream of the RT was strongly enriched by RIP-seq, but in contrast to DRT2, cDIP-seq analysis failed to detect a reverse transcription product (Fig. 1c,d). Given the genetic evidence that reverse transcription activity is essential for phage defense (Extended Data Fig. 1d), we reasoned that DRT9 indeed produces a cDNA, though its identification might require an alternative approach.

**Extended Data Figure 2 |:**
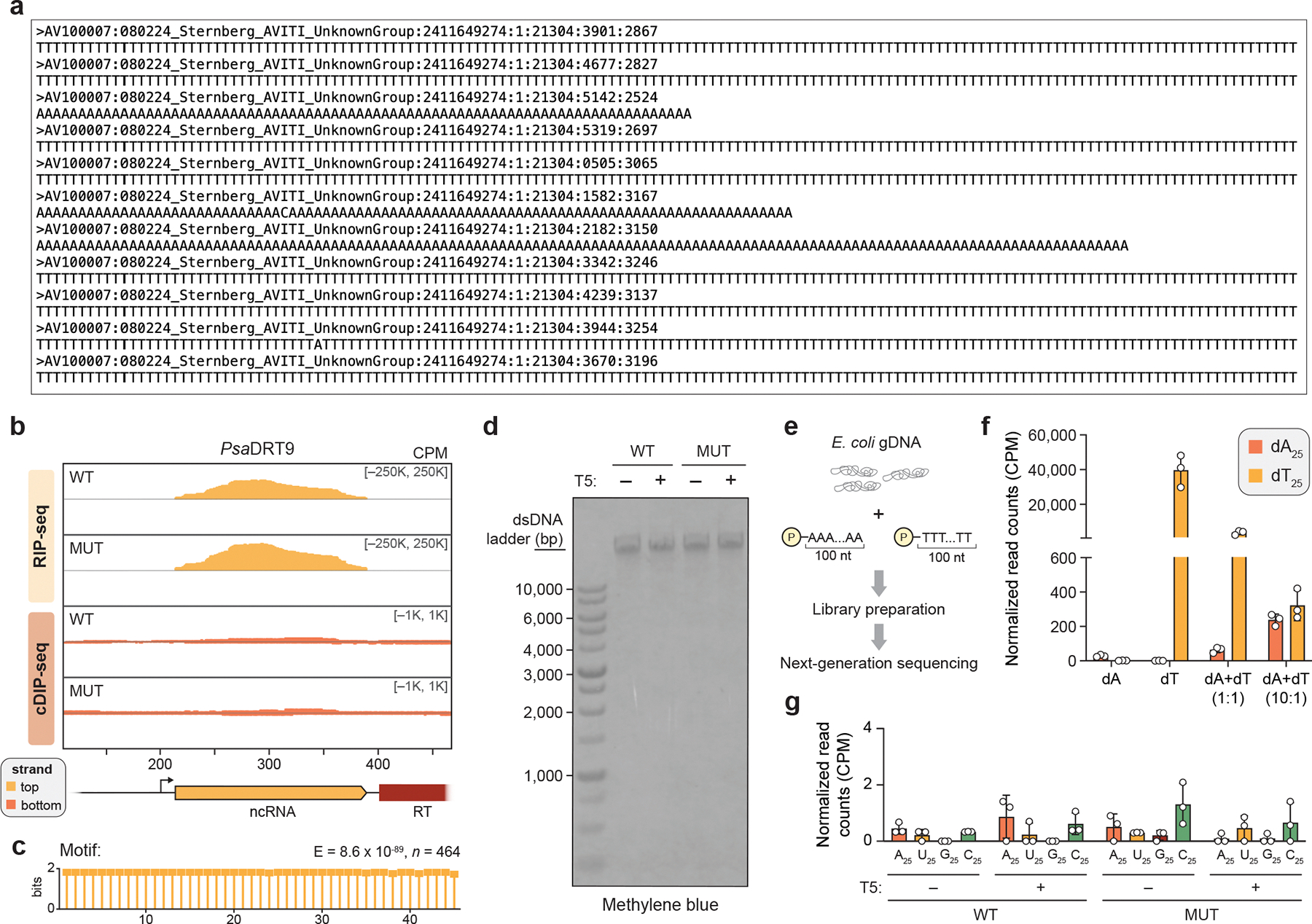
Discovery and characterization of homopolymeric DNA products elicited by DRT9 immune systems. **a,** Unmapped reads from a cDIP-seq dataset of *Sen*DRT9-expressing cells infected with T5 phage, showing uninterrupted strings of poly-dT and poly-dA. **b,** RIP-seq and cDIP-seq coverage tracks (top to bottom) for either WT or RT-inactive (MUT) *Psa*DRT9, in the presence of T5 phage infection. A schematic of the genomic locus is shown below; data are normalized for sequencing depth and plotted as counts per million reads (CPM). **c,** MEME analysis results revealing a poly-dT motif enriched in unmapped reads from the WT + T5 cDIP-seq dataset in **b**. *E*, E-value significance; *n*, number of contributing sites. **d,** Methylene blue-stained membrane used for the Southern blot shown in Fig. 1h. Representative data are shown for experiments repeated at least two times with similar results. **e,** Schematic of oligo spike-in experiment to address potential bias in next-generation sequencing-based detection of poly-dA and poly-dT on the AVITI platform; P, phosphorylated 5′ end. **f,** Bar graph of dA_25_ and dT_25_ counts from total DNA sequencing of oligo spike-in experiments schematized in **e**; the apparent bias against poly-dA capture and sequencing leads to artificially elevated levels of dT_25_-containing reads relative to dA_25_-containing reads. **g,** Bar graph of normalized homopolymer counts from total RNA-seq datasets of WT and MUT *Sen*DRT9-expressing cells +/− T5 phage infection. These data demonstrate that poly-dA and poly-dT cDNAs are not transcribed, in contrast to the cDNA products of DRT2. Data in **f,g** are shown as mean ± s.d. for n = 3 independent biological replicates.

Inspired by reports that AbiK RTs synthesize untemplated, random DNA polymers^14,16^, we hypothesized that analyzing cDIP-seq reads that failed to map to the reference genome might reveal unexpected cDNA products. Inspection of alignment statistics revealed a striking and specific increase in unmapped reads for phage-infected samples expressing WT *Sen*RT (Fig. 1e). This observation prompted us to manually inspect the unmapped reads, which uncovered an unanticipated result: long, uninterrupted stretches of poly-deoxythymidylate (poly-dT) and poly-deoxyadenylate (poly-dA), a pattern further corroborated by motif detection analysis (Fig. 1f, Extended Data Fig. 2a). RIP-seq and cDIP-seq experiments with the related *Psa*DRT9 system similarly revealed strong enrichment of the upstream ncRNA, an absence of cDNA species mapping to the ncRNA, and a significant overrepresentation of poly-dT motifs within unmapped reads (Extended Data Fig. 2b,c). These results provide the first evidence of an RT naturally synthesizing a DNA homopolymer and implicate poly-dT and/or poly-dA products in the mechanism of antiphage defense.

We next quantified reads containing homopolymers (≥25 nucleotides in length) of each of the four deoxynucleotides across all cDIP-seq datasets. Poly-dT and poly-dA were detected exclusively in phage-infected cells expressing WT *Sen*DRT9, with poly-dT outnumbering poly-dA by a factor of ~10, while poly-dG and poly-dC products were not detectable above background levels (Fig. 1g). To account for potential sequencing artifacts affecting homopolymer detection and quantification, we turned to a classical molecular biology approach to independently assess poly-dT and poly-dA production. Southern blotting experiments using oligo-dT and oligo-dA probes corroborated our cDIP-seq results, revealing abundant poly-dA and poly-dT species, respectively, that were again restricted to phage-infected cells expressing WT *Sen*DRT9 (Fig. 1h, Extended Data Fig. 2d). Two additional observations from Southern blotting were notable. First, poly-dA and poly-dT species ranged from ~1,000 to >10,000 bases in length, relative to a double-stranded DNA (dsDNA) ladder (Fig. 1h). Second, poly-dA products were substantially more abundant than poly-dT products by Southern blotting, in contrast to the relative proportions observed by sequencing (Fig. 1g,h). To investigate this discrepancy, we performed control experiments in which synthetic dT_100_ and dA_100_ oligonucleotides were spiked into *E. coli* cell lysates at varying ratios prior to library preparation and sequencing (Extended Data Fig. 2e). These experiments revealed a strong bias against poly-dA capture and sequencing relative to poly-dT (Extended Data Fig. 2f), suggesting that Southern blot data more accurately reflect the true intracellular distribution of homopolymer products. High-throughput sequencing nevertheless provided insights that would have been challenging to detect otherwise, including the presence of chimeric species harboring dA_10_–dT_10_ transition sequences (Fig. 1i). Interestingly, these species were more abundant than their inverse counterparts (dT_10_–dA_10_), suggesting that initial cDNA synthesis by DRT9 may exclusively produce poly-dA, which occasionally serves as a template and a primer to generate a complementary poly-dT strand.

Collectively, these findings indicate that phage infection triggers the synthesis of poly-dA by the DRT9 immune system, while poly-dT likely arises from low-level second-strand synthesis by the RT itself or another DNA polymerase. Of note, RNA-seq datasets did not reveal any evidence of poly-A or poly-U species (Extended Data Fig. 2g), thus arguing against any coding role for DRT9 cDNA synthesis — a notable divergence from DRT2. This observation, along with the distinct nature of the cDNA sequence, underscores the functional and molecular diversity of DRT enzymes and their reverse transcription products.

## The DRT9 ncRNA templates poly-dA synthesis

**Extended Data Figure 3 |:**
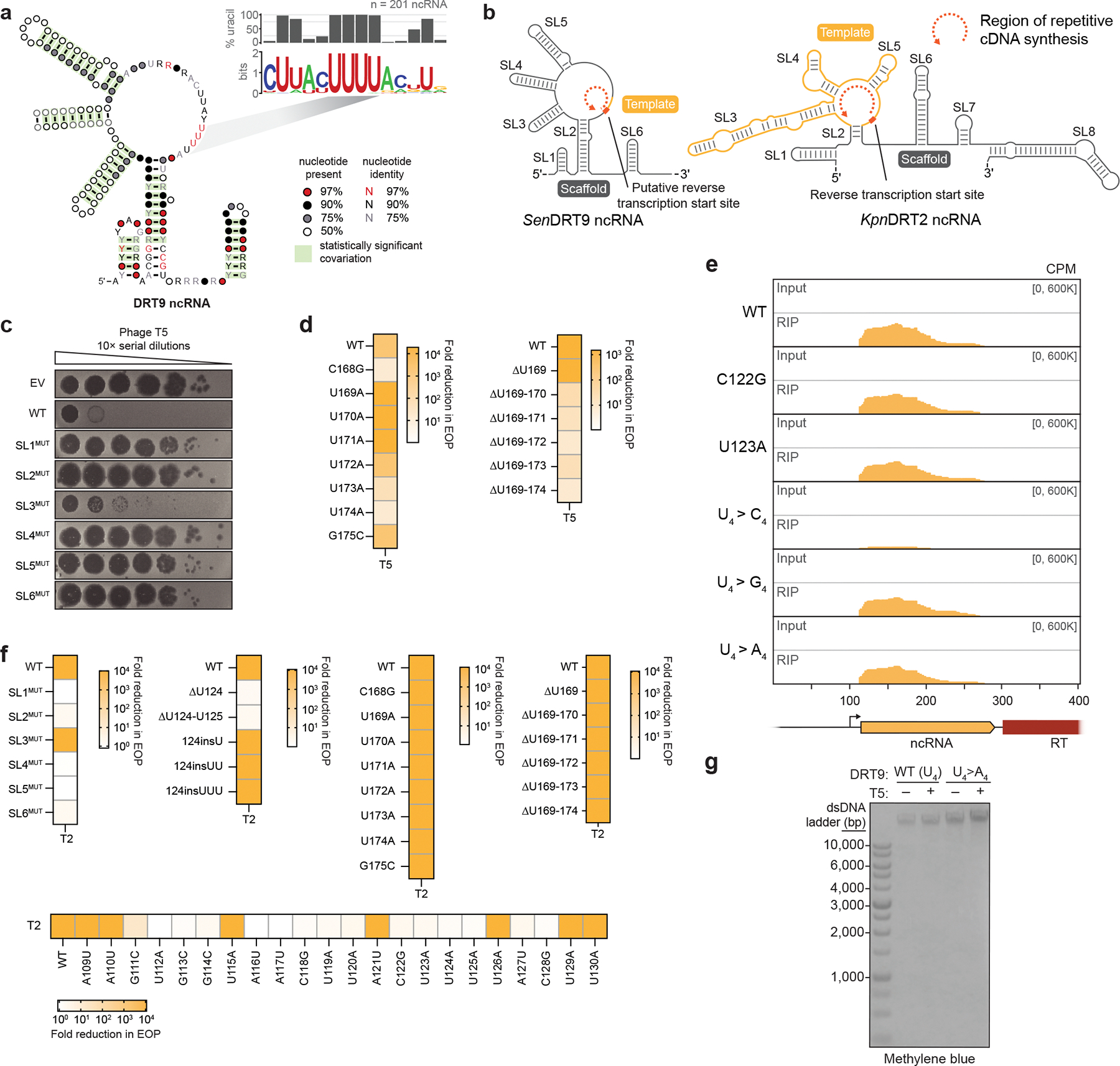
Additional characterization of ncRNA sequence perturbations on *Sen*DRT9 defense activity. **a,** Covariance model of the DRT9 ncRNA from an analysis of 201 homologous systems (left), and WebLogo from a multiple sequence alignment centered around the putative U-rich template region (top right). **b,** Comparison of *Sen*DRT9 (left) and *Kpn*DRT2 (right) ncRNAs, highlighting the scaffold (grey) and template (orange) regions. Both ncRNAs template cDNA synthesis from a similar location relative to SL2 (reverse transcription start site, in red), and program repetitive cDNA synthesis across the template region. **c,** Representative plaque assays for the data shown in Fig. 2b. **d,** Heat map quantifying *Sen*DRT9 defense activity against T5 phage for the indicated ncRNA mutations and deletions within the 3′-proximal SL6 and U-rich region, quantified as the fold reduction in EOP relative to an empty vector (EV) control; data are shown as the mean of n = 2 technical replicates. **e,** RIP-seq coverage tracks for *Sen*DRT9 with WT ncRNA or the indicated ncRNA mutations in uninfected cells; the bottom three variants are mutated in the putative template region (residues 123–126). A schematic of the genomic locus is shown below; data are normalized for sequencing depth and plotted as counts per million reads (CPM). **f,** Heat map quantifying *Sen*DRT9 defense activity against T2 phage for the same ncRNA mutations tested against T5 phage in Fig. 2b–d and panel **d**; data are shown as in **d**. **g,** Methylene blue-stained membrane used for the Southern blot shown in Fig. 2f. Representative data are shown for experiments repeated at least two times with similar results.

**Figure 2 |:**
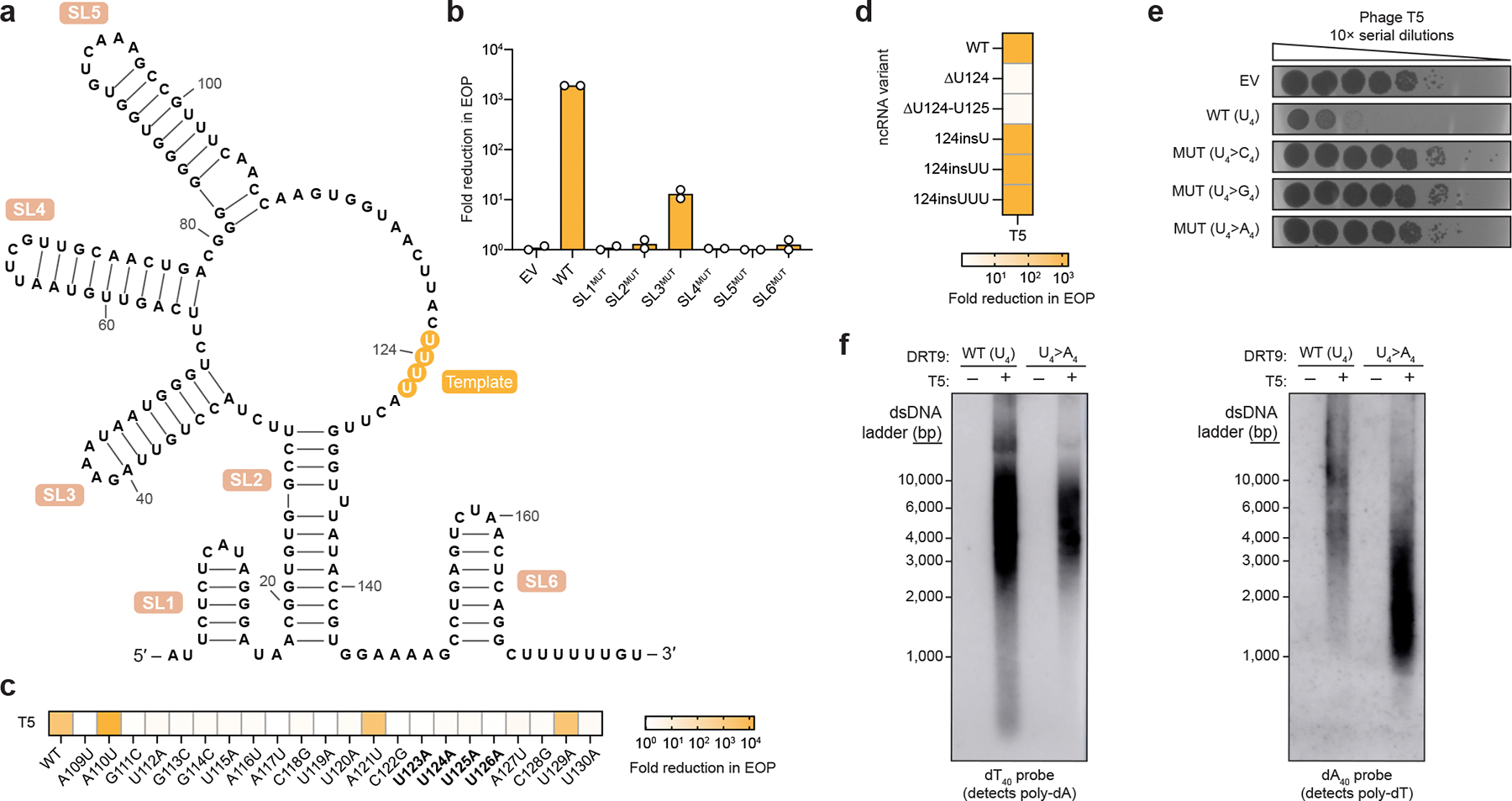
ncRNA sequence determinants of *Sen*DRT9-mediated phage defense and poly-dA synthesis. **a,** Predicted secondary structure of the *Sen*DRT9 ncRNA. Stem-loop (SL) regions and uridine nucleotides implicated in RNA-templated DNA synthesis are labeled; coordinates are numbered based on the mature ncRNA species identified by RIP-seq. **b,** Bar graph quantifying *Sen*DRT9 defense activity against T5 phage for scrambled ncRNA SL mutants (MUT), quantified as the fold reduction in efficiency of plating (EOP) relative to an empty vector (EV) control. Data are from n = 2 technical replicates. **c,** Heat map quantifying *Sen*DRT9 defense activity for the indicated ncRNA point mutations, quantified as in **b**. Uridine nucleotides implicated in RNA-templated DNA synthesis are highlighted in bold; data are shown as the mean of n = 2 technical replicates. **d,** Heat map quantifying *Sen*DRT9 defense activity for the indicated ncRNA mutations, shown as in **c**. **e,** Plaque assay showing loss of *Sen*DRT9 defense activity against T5 phage for substitutions within the putative ncRNA template region; residues U123–U126 (U_4_) were mutated to each of the indicated nucleotides. **f,** Southern blot analysis of total DNA isolated from cells expressing the WT or U_4_>A_4_ MUT ncRNA from **e**, +/− T5 phage infection. Duplicate blots were probed with either oligo-dT_40_ (left) or oligo-dA_40_ (right); sizes from a dsDNA ladder are marked. Representative data are shown for experiments repeated at least two times with similar results.

We set out to examine how an RT synthesizes kilobase-length polymers composed almost exclusively of adenosine nucleotides. We initially considered the hypothesis that the RT might directly select dATP substrates through protein-mediated recognition rather than using a nucleic acid template, akin to the reported mechanism of untemplated DNA synthesis by AbiK^16^. However, closer inspection of the DRT9 ncRNA sequence and predicted secondary structure suggested an alternative hypothesis. In addition to conserved stem-loop (SL) elements that loosely resemble the overall architecture of DRT2 ncRNAs, DRT9 ncRNAs contain a universally conserved uridine-rich stretch positioned similarly to the DRT2 reverse transcription start site (Fig. 2a, Extended Data Fig. 3a,b). We envisioned that this stretch of uridines could serve as a template for poly-dA synthesis, potentially via iterative synthesis, melting, and realignment of the growing poly-dA strand across from complementary uridine nucleotides.

To investigate the potential structural and templating functions of the ncRNA, we scrambled each of the conserved SL regions and assessed the effect of SL perturbation on antiphage defense activity. All SLs were essential for defense against T5 phage except for SL3, the disruption of which had a mild effect (Fig. 2b, Extended Data Fig. 3c). We then introduced single-nucleotide substitutions within a prominent loop region containing the putative U-rich template. Most mutations in this loop severely impaired defense, suggesting a critical role for this region in ncRNA function (Fig. 2c). Strikingly, deletion of a single uridine residue (U124) completely abolished defense, whereas similar perturbations in a secondary U-rich region at the 3′ end of the ncRNA had no effect (Fig. 2a,d, Extended Data Fig. 3d). Importantly, RIP-seq experiments on a subset of ncRNA mutants revealed that the observed loss of defense activity was in most cases not attributable to defects in RT-ncRNA binding (Extended Data Fig. 3e). The effects of these ncRNA perturbations tested against T2 phage closely mirrored those observed with T5 (Extended Data Fig. 3f), further supporting the functional importance of ncRNA structural and sequence motifs.

We further investigated the putative templating role of the ncRNA by testing additional mutations to the U-rich region. Mutating residues 123–126 (U_4_) to alternative nucleotides (C_4_, G_4_, or A_4_) abolished phage defense in all cases (Fig. 2e), initially suggesting loss of DNA synthesis with these mutants. However, Southern blot analysis of the U_4_>A_4_ mutant revealed an inversion of the WT homopolymer distribution: instead of primarily producing poly-dA with lower levels of poly-dT, the A_4_ template primarily generated poly-dT with lower levels of poly-dA (Fig. 2f, Extended Data Fig. 3g). Technical challenges related to the chemical synthesis of guanosine- and cytidine-only oligonucleotides prevented us from testing whether the U_4_>G_4_ and U_4_>C_4_ variants could similarly template complementary homopolymers by Southern blotting.

These results implicate the U-rich loop as an RNA template for cDNA synthesis. Like DRT2 systems, which perform rolling circle reverse transcription of a ~120-nt template to generate repetitive cDNA products^4,5^, *Sen*DRT9 also spools out repetitive cDNA products. However, unlike DRT2, *Sen*DRT9 templates a single nucleotide identity, yielding uninterrupted homopolymers.

## DRT9 protein priming and oligomerization

**Extended Data Figure 4 |:**
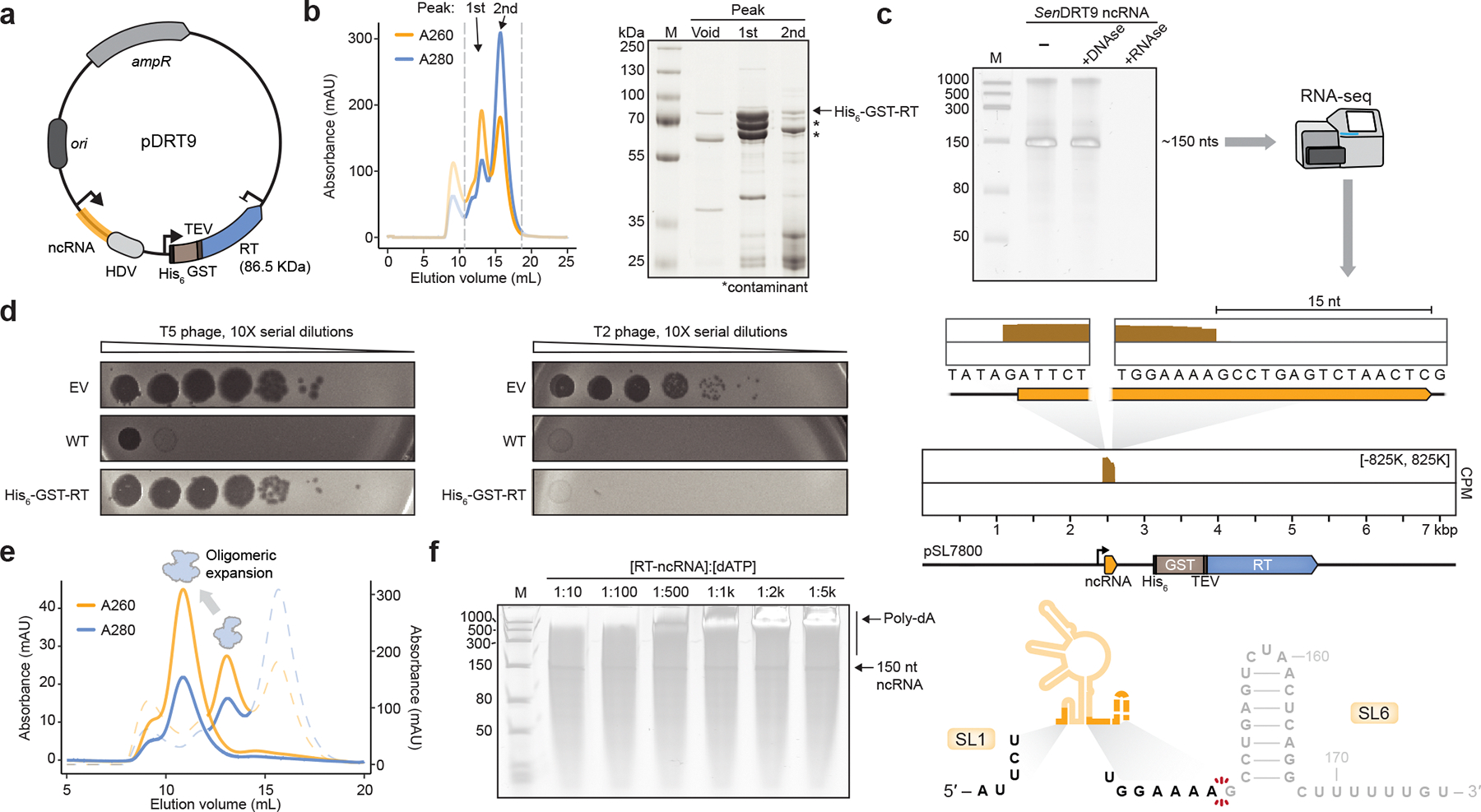
Purification and characterization of the *Sen*DRT9-encoded RT-ncRNA complex. **a,** *E. coli* expression vector design for the *Sen*DRT9-encoded ncRNA and His_6_-GST-tagged RT. **b,** Size-exclusion chromatogram of the His_6_-GST-tagged RT-ncRNA complex separated on a Superdex 200 10/300 column (left), and SDS-PAGE analysis of the void volume and labeled peaks (right); the high A_260_:A_280_ ratio is consistent with a protein-nucleic acid complex. **c,** Denaturing, SYBR Gold-stained 10% urea-PAGE analysis of the ~150-nt ncRNA species co-purifying with *Sen*RT after RNase or DNase treatment (top), and RNA-seq analysis of the ncRNA (middle). The mature ncRNA carries an extraneous 5′-G resulting from the T7 promoter and lacks SL6 at the 3′ end (bottom), suggesting that SL6 may be involved in transcriptional termination *in vivo* while being dispensable for RT-mediated poly-dA synthesis. **d,** Plaque assay showing loss of *Sen*DRT9 defense activity for a His_6_-GST-tagged RT variant against T5 phage (left), but not T2 phage (right); EV, empty vector. **e,** Overlaid chromatograms from gel filtration experiments with RT-ncRNA complexes before and after TEV protease treatment of the His_6_-GST-RT fusion protein, revealing a shift to earlier retention volume and thus increased oligomeric state; the persistent high A_260_:A_280_ ratio suggests that the RT-ncRNA interaction remains intact. **f,** Denaturing 8% urea-PAGE analysis of DNA polymerization assays that contained 150 nM RT-ncRNA complex and increasing amounts of dATP per RT monomer, as indicated. Reactions were incubated at 37 °C for 60 min, followed by proteinase K treatment and phenol-chloroform extraction prior to electrophoretic separation; the gel was stained with SYBR Gold. For **b,c,f,** representative data are shown for experiments repeated at least two times with similar results.

**Figure 3 |:**
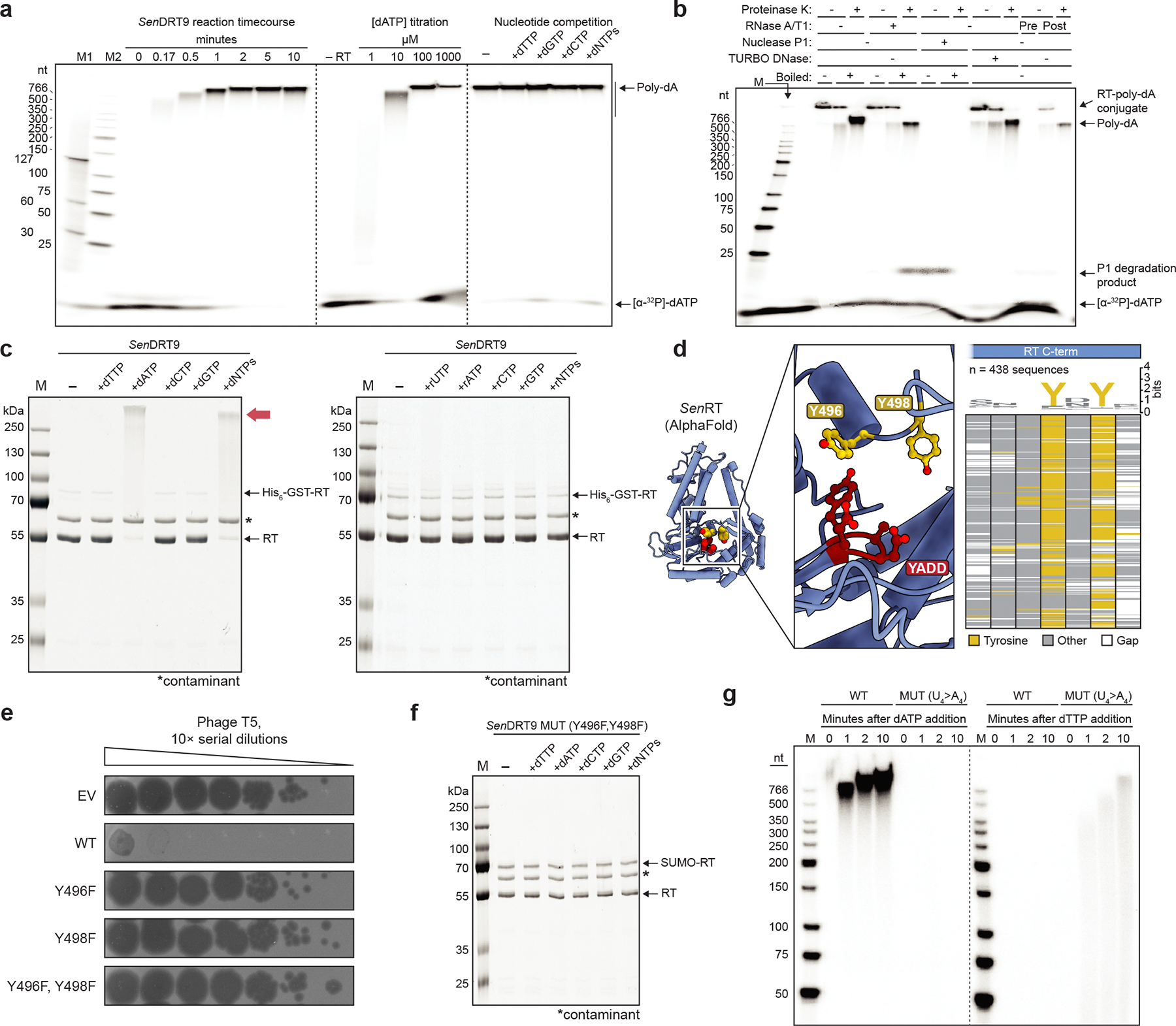
The *Sen*DRT9 RT-ncRNA complex performs protein-primed, RNA-templated DNA homopolymer synthesis. **a,** Denaturing 5% urea-PAGE analysis of DNA polymerization assays testing the effects of time (left), dATP concentration (middle), and nucleotide competition (right) on poly-dA synthesis. All reactions contained 20 nM RT-ncRNA complex; *Left*, reactions contained 100 μM dATP; *Middle*, reactions were incubated for 10 min; *Right*, reactions were incubated for 10 min with 100 μM radiolabeled dATP and unlabeled nucleotides at 100 μM. M1 and M2 denote DNA ladder markers. **b,** Denaturing 5% urea-PAGE analysis of DNA polymerization assays, after reactions were treated with the indicated proteinase or nuclease reagents +/− boiling. All reactions contained 20 nM RT-ncRNA and 100 μM [α-^32^P]-dATP, and were incubated at 37 °C for 10 min prior to proteinase/nuclease addition, with the exception of lane 15, which was pre-treated with RNase A/T1 prior to dATP addition. **c,** SDS-PAGE analysis of DNA polymerization assays, in which RT-ncRNA complexes (0.15 μM) were incubated with the indicated dNTPs or rNTPs (0.9 mM) for 60 min at 37 °C. The red arrow denotes high-molecular weight (MW) protein-DNA conjugates in reactions that contained dATP; the His_6_-GST-RT band arises from incomplete tag removal; * purification contaminant; M, protein marker. **d,** *Sen*RT AlphaFold 3 structure prediction (left), with magnified view (middle) showing the close proximity of C-terminal residues Y496 and Y498 (yellow) to the YADD active site (red); the multiple sequence alignment (right) highlights their strong conservation. **e,** Plaque assay showing loss of *Sen*DRT9 defense activity against T5 phage for the indicated mutants; EV, empty vector. **f,** SDS-PAGE analysis of DNA polymerization assays as in **c**, but with a Y496F,Y498F RT mutant. **g,** Denaturing 5% urea-PAGE analysis of DNA polymerization assays with either WT or U4>A4 mutant ncRNA in the presence of [α-^32^P]-dATP (left) or [α-^32^P]-dTTP (right). All reactions contained 20 nM RT-ncRNA complexes and 100 μM [α-^32^P]-dATP or [α-^32^P]-dTTP. For **a-c,f,g,** representative data are shown for experiments repeated at least two times with similar results.

We next sought to determine the biochemical basis for RNA-templated DNA homopolymer synthesis by DRT9. We initially purified *Sen*RT as a His_6_-GST fusion protein, which co-purified with an RNA species corresponding to the first ~150 nt of the full-length ncRNA transcript (Extended Data Fig. 4a–c). Given that His_6_-GST-tagged RT constructs exhibited reduced phage defense activity (Extended Data Fig. 4d), we reasoned that the affinity tag and solubilization domain might impair RT activity. Interestingly, after protease cleavage of the N-terminal tag, we observed a shift in the gel filtration retention volume to a monodisperse, higher molecular weight (MW) species, consistent with oligomerization (Extended Data Fig. 4e). Incubation of the oligomeric RT-ncRNA complex with [α-^32^P]-dATP, followed by denaturing urea-PAGE analysis, revealed robust and highly processive poly-dA synthesis activity that completely consumed dATP in the reaction, and that required no additional reaction components beyond the RT and ncRNA (Fig. 3a). Poly-dA products were 100s to >1000 nt in length and insensitive to the addition of alternative nucleotide substrates (Fig. 3a, Extended Data Fig. 4f), corroborating the conclusions from cDIP-seq and Southern blot analyses that DRT9 produces long, homopolymeric DNA.

**Extended Data Figure 5 |:**
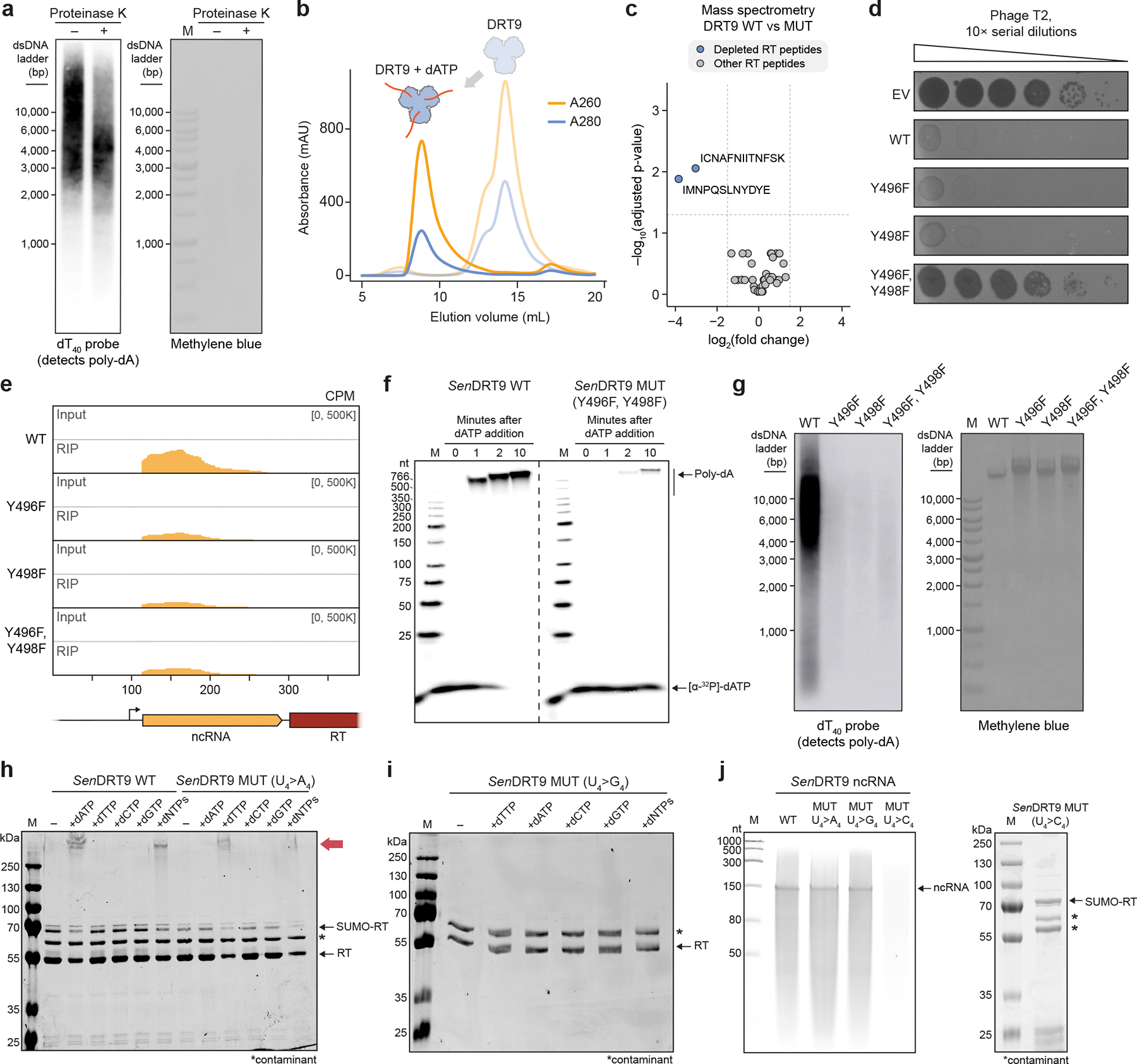
Roles of C-terminal tyrosine residues in protein-primed reverse transcription and DRT9 phage defense. **a,** Southern blot analysis of total DNA isolated from T5-infected cells expressing WT *Sen*DRT9 +/− proteinase K treatment. The blot was probed with oligo-dT_40_ (left) to detect poly-dA species; the methylene blue-stained membrane after transfer is shown at right; M denotes a ladder marker. **b**, Overlaid chromatograms from gel filtration analysis of RT-ncRNA complexes +/− dATP incubation, revealing an increased A_260_:A_280_ ratio and a dramatic shift in retention volume in the presence of dATP. **c**, Volcano plot of differential peptide abundance from mass spectrometry analysis of WT versus catalytically inactive (MUT) RT proteins immunoprecipitated from T5-infected cells expressing *Sen*DRT9. Each dot indicates a *Sen*RT peptide, and blue dots indicate significantly depleted peptides with log_2_(fold change) < −1.5 and adjusted p-value < 0.05, as determined by unpaired two-tailed t-test with correction for multiple comparisons using the Benjamini-Hochberg method; the most depleted peptide, IMNPQSLNYDYE, contains two tyrosine residues likely involved in priming of poly-dA synthesis. **d,** Plaque assay showing loss of *Sen*DRT9 defense activity against T2 phage for a double Y496F,Y498F mutant, but not single Y496F or Y498F mutants; EV, empty vector. **e,** RIP-seq coverage tracks for *Sen*DRT9 in T5-infected cells with WT and single or double Y496F/Y498F RT mutants, as indicated. A schematic of the genomic locus is shown below; data are normalized for sequencing depth and plotted as counts per million reads (CPM). **f,** Denaturing 5% urea-PAGE analysis of DNA polymerization assays with WT (left) and Y496F,Y498F mutant (right) RT. All reactions contained 20 nM RT-ncRNA and 100 μM [α-^32^P]-dATP; M denotes a DNA ladder marker. **g,** Southern blot analysis of total DNA isolated from T5-infected cells expressing *Sen*DRT9 with WT and single or double Y496F/Y498F RT mutants, as indicated. The blot was probed with oligo-dT_40_ (left) to detect poly-dA species; the methylene blue-stained membrane after transfer is shown at right; M denotes a DNA ladder marker. **h**, SDS-PAGE analysis of DNA polymerization assays with either WT *Sen*DRT9 or a U_4_>A_4_ mutant ncRNA. RT-ncRNA complexes (0.15 μM) were incubated with the indicated dNTPs (0.9 mM). The red arrow denotes high-molecular weight (MW) protein-DNA conjugates; the SUMO-RT band arises from incomplete tag removal; *, purification contaminant; M, protein ladder marker. **i**, SDS-PAGE analysis of DNA polymerization assays as in **h**, but with a U_4_>G_4_ mutant ncRNA; no high-MW species are observed. **j**, Denaturing, SYBR Gold-stained 10% urea-PAGE analysis (left) of WT, U_4_>A_4_, and U_4_>G_4_ ncRNAs co-purifying with *Sen*RT. The absence of a band for the U_4_>C_4_ ncRNA, despite a clear band corresponding to the RT by SDS-PAGE (right), indicates that the U_4_>C_4_ mutation disrupts RT-ncRNA complex formation. For **a,f-j,** representative data are shown for experiments repeated at least two times with similar results.

Additional observations from analyses of RT reaction products provided insights into the mechanism of homopolymer synthesis. Single-strand-specific Nuclease P1 completely degraded poly-dA, whereas TURBO DNase — which preferentially degrades dsDNA — had little effect (Fig. 3b). Meanwhile, proteinase K treatment altered the mobility of poly-dA, whereas RNase treatment had no effect (Fig. 3b). A similar effect on mobility was observed with Southern blotting of proteinase K-treated poly-dA products isolated from cells (Extended Data Fig. 5a). Strikingly, when RT-ncRNA complexes were incubated with unlabeled dNTP/NTP substrates and then analyzed by SDS-PAGE, we observed a dramatic mobility shift of the RT protein in reactions that contained dATP (Fig. 3c). Consistent with these results, gel filtration analysis of the RT-ncRNA complex following dATP incubation revealed conversion into a distinct species that exhibited a heightened A_260_/A_280_ ratio and ran in the void volume, indicating increased nucleic acid content and molecular weight (Extended Data Fig. 5b). Taken together, these results suggest that poly-dA products are covalently linked to the RT protein itself, potentially via priming by a hydroxyl-containing amino acid side chain.

To identify candidate RT residues involved in protein priming of poly-dA synthesis, we immunoprecipitated the WT or catalytically inactive (MUT) RT from T5-infected cells and analyzed RT peptides by mass spectrometry. We hypothesized that a covalent DNA linkage would interfere with peptide detection, leading to apparent depletion of any poly-dA-linked peptide. Strikingly, only two peptides were significantly depleted in WT samples, relative to the MUT condition (Extended Data Fig. 5c). Closer inspection of the AlphaFold-predicted RT monomer structure revealed that one of these peptides, derived from the C-terminal tail, contains two highly conserved tyrosine residues positioned in the immediate vicinity of the active site (Fig. 3d). Given the established role of tyrosine residues in protein-primed DNA synthesis by other RTs ^16,18,19^, we hypothesized that one or both of these tyrosine residues could similarly serve as primers for reverse transcription by the DRT9 RT.

To test this hypothesis, we individually or simultaneously mutated each of the putative priming tyrosines to phenylalanine and assessed the effect on DRT9 activity. Phage defense against T5 was completely abolished when either tyrosine residue was mutated (Fig. 3e). In contrast, loss of defense against T2 required substitution of both tyrosines (Extended Data Fig. 5d), suggesting that either residue can serve as a priming site. RIP-seq experiments in T5-infected cells showed that Tyr-to-Phe (Tyr>Phe) RT mutants exhibited mildly decreased ncRNA binding relative to the WT RT (Extended Data Fig. 5e), suggesting that the observed loss of defense may have also resulted in part from weakened RT-ncRNA interactions. Nonetheless, both *in vitro* polymerization reactions and *in vivo* experiments with phage-infected cells showed severe attenuation of poly-dA synthesis with the double Tyr>Phe mutant (Fig. 3f, Extended Data Fig. 5f,g). The residual presence of low levels of poly-dA product suggests that additional hydroxyl-containing residues in the RT active site could perhaps be used as alternative primers, albeit with considerably reduced efficiencies. Altogether, these results implicate C-terminal tyrosines within the *Sen*DRT9 RT as primers for poly-dA synthesis.

Finally, we sought to further assess our model for uridine templating of poly-dA synthesis through *in vitro* biochemical experiments. We purified RT-ncRNA complexes containing the same U_4_>A_4_ substitution tested in phage defense assays and assessed their polymerization activity. Whereas the WT RT-ncRNA complex efficiently polymerized dATP, but not dTTP, the opposite substrate preference was observed with the U_4_>A_4_ ncRNA mutant (Fig. 3g, Extended Data Fig. 5h). Similar experiments using a U_4_>G_4_ mutant did not show evidence of dCTP polymerization (Extended Data Fig. 5i), and the U_4_>C_4_ ncRNA failed to co-purify with the RT (Extended Data Fig. 5j), consistent with our earlier RIP-seq results (Extended Data Fig. 3e). These data indicate that templated homopolymer synthesis by DRT9 is reprogrammable, albeit limited to certain template sequences, potentially reflecting a combination of factors that could include ncRNA folding, base pairing energetics for repeated rounds of polymerization and melting, and structural flexibility of the homopolymer product.

## Cryo-EM structure of the *Sen*DRT9 complex

**Extended Data Figure 6 |:**
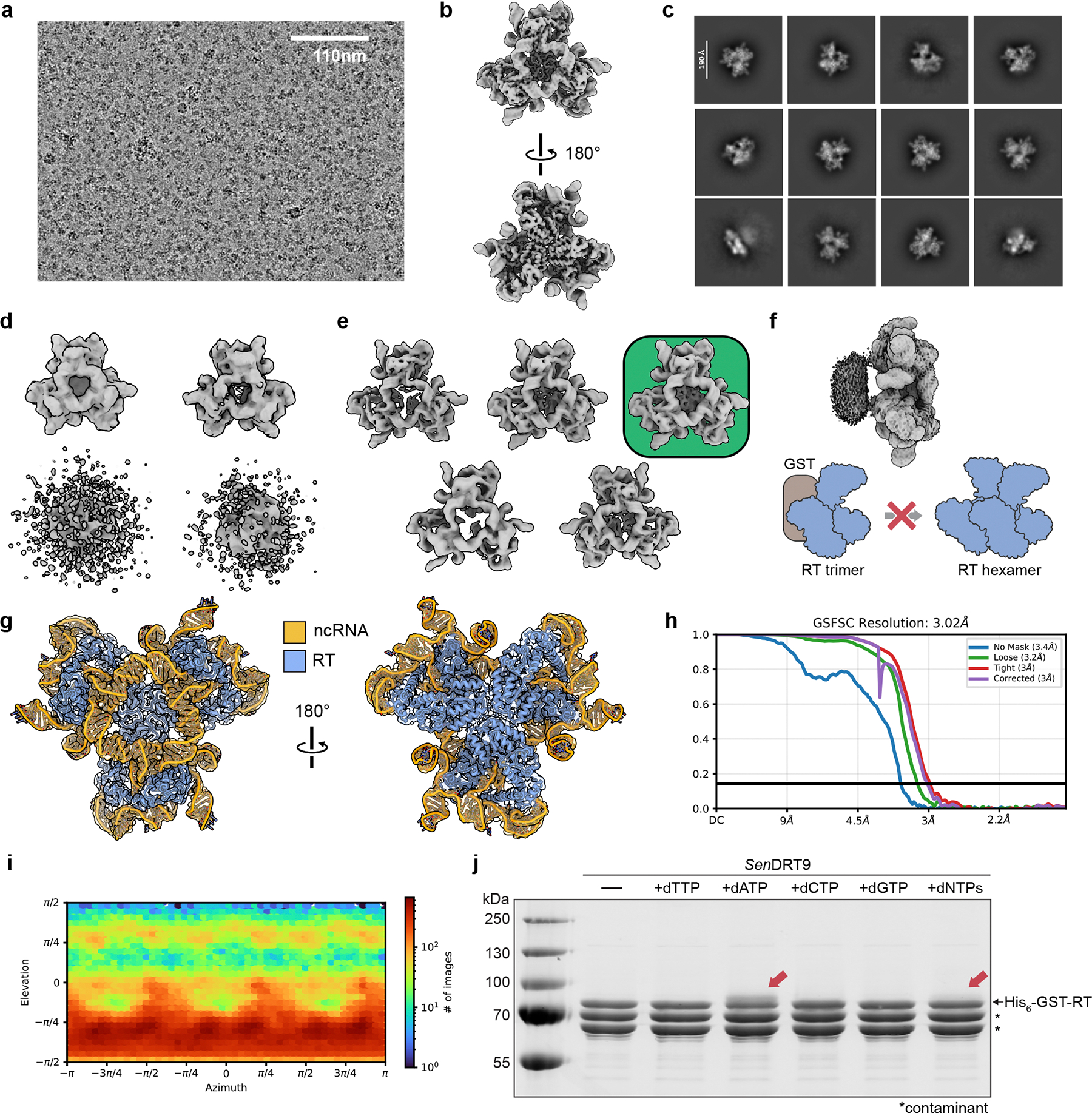
Cryo-EM analysis and image processing workflow for trimeric *Sen*DRT9 RT-ncRNA complex. **a,** Representative micrograph from *Sen*DRT9 RT-ncRNA complex data collection with the His_6_-GST fusion protein, revealing a densely-packed field of particles in vitreous ice. **b,** Initial consensus volume after processing blob-picked particles, which refined to 3.7 Å resolution (C3-reconstruction) for *de novo* template generation and subsequent particle picking. **c,** Representative selected 2D classes from 5,235,086 initial particles picked using the template in panel **b**. **d,** Four-class *ab initio* reconstruction from 2,530,970 particles in selected classes to remove junk particles. **e,** Results of a five-class 3D classification without a mask and filtered at 8 Å resolution, with a class similarity of 0.1 to remove remaining poorly aligned particles. The best volume, highlighted by a green background, contained 367,640 particles that were selected for further processing. **f,** View of the final reconstruction in **g** shown at high contour (top), revealing noisy density at the N-terminus of each RT monomer corresponding to the His_6_-GST fusion used for expression and purification. We hypothesized that the GST fusion would prevent the formation of higher-order oligomers (bottom). **g,** Final (C3) reconstruction that refined to 3 Å resolution and was used for building the trimeric RT-ncRNA complex model. The map is shown with partial transparency and colored according to the model, with RT subunits in blue and ncRNA subunits in orange. **h,** Plot of Cryosparc’s gold standard Fourier shell correlation for the map in **g**. **i,** Plot of particle view distribution reveals mild anisotropy but no missing views. **j,** SDS-PAGE analysis of DNA polymerization assays with His_6_-GST-RT sample, in which RT-ncRNA complexes were incubated with the indicated dNTP(s) before reactions were quenched and resolved electrophoretically. Red arrows indicate preliminary evidence of protein-DNA conjugates in reactions that contained dATP. Representative data are shown for experiments repeated at least two times with similar results.

**Figure 4 |:**
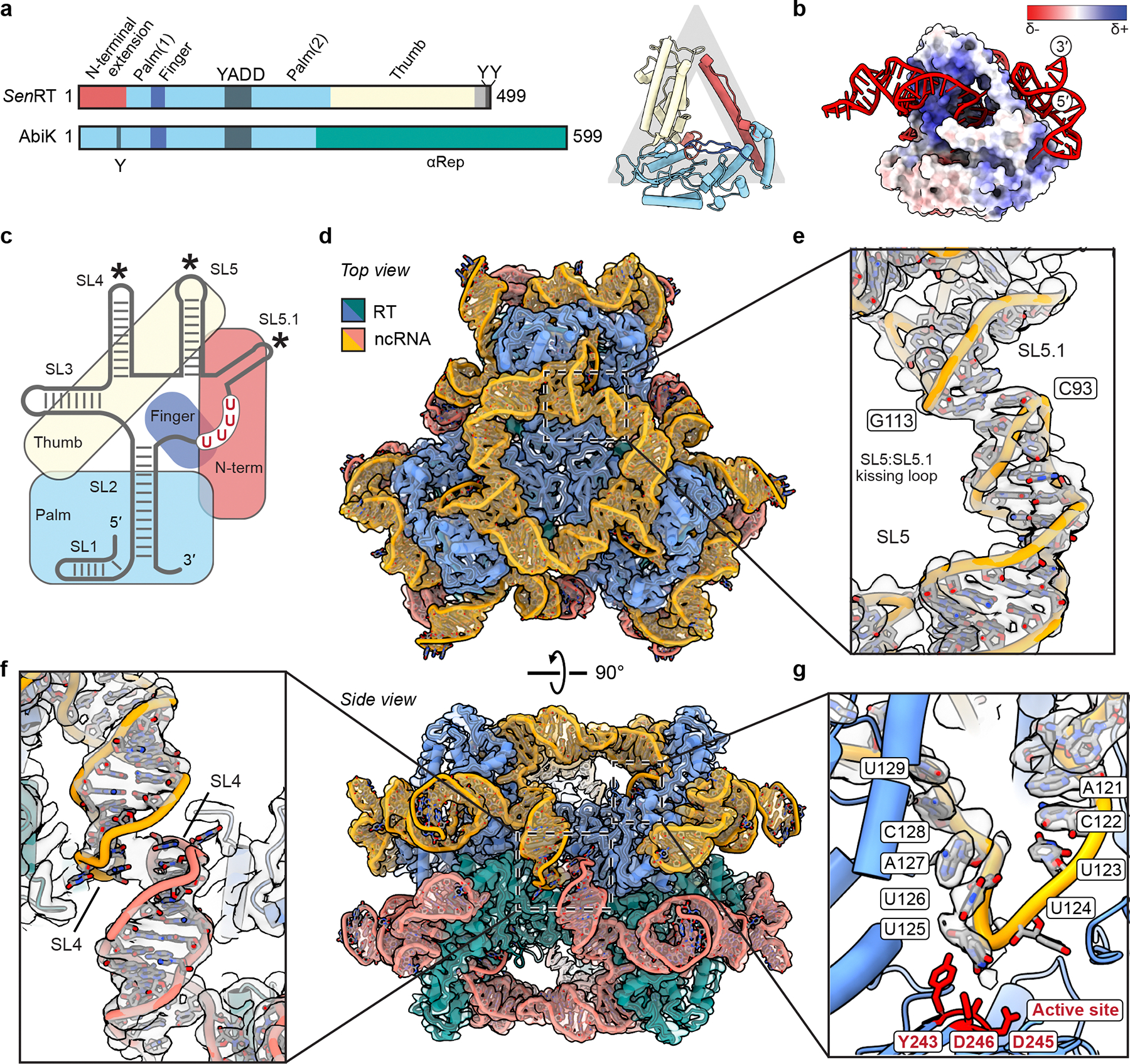
Cryo-EM structure of the hexameric *Sen*DRT9 RT-ncRNA complex. **a,** Domain architecture (left) of *Sen*DRT9 RT (PDB: 9NLX, this study) compared to AbiK (PDB: 7R06); the *Sen*RT has an N-terminal extension (red) that reaches toward the thumb domain (right), creating a triangular architecture. **b,** Surface representation of a single RT monomer colored by relative electrostatic potential; the 3′ end of the ncRNA (red ribbon) threads through the center of the RT triangle. **c,** Cartoon representation of the ncRNA secondary structure observed in the cryo-EM density, with features involved in inter-subunit interactions highlighted with asterisks. The ncRNA interacts with each domain of the RT, represented as rectangles colored as in **a**. **d,** A 2.6 Å semi-transparent map and resulting model of the hexameric *Sen*DRT9 RT-ncRNA complex (PDB: 9NLV, this study). The top view looks down the three-fold symmetric axis of one trimer, and the side view reveals the back-to-back arrangement of two trimers. RTs and ncRNAs in the two trimers are colored in blue/green and yellow/salmon, respectively. **e,** Semi-transparent density map and model of the kissing loop interactions between flipped out bases in SL5 (C93) and SL5.1 (G113) of adjacent ncRNAs. **f,** Semi-transparent density map and model showing tetraloops in SL4. **g,** A close-up view of the templating nucleotides U123-U126 and the polymerase active site (red, YADD); density for the RT is hidden for simplicity.

To better understand the molecular and structural basis for poly-dA synthesis by *Sen*DRT9, we determined the structure of the His_6_-GST-tagged RT and ncRNA using cryogenic electron microscopy (cryo-EM) (Extended Data Fig. 6a–i). A 3.0 Å-resolution reconstruction enabled unambiguous assignment of 98% of the residues in the RT and 85% of nucleotides in the ncRNA (Extended Data Table 1). The RT contains the three characteristic finger, thumb, and palm domains of canonical reverse transcriptases, and an N-terminal extension (residues 1–58) reaches towards the C-terminal thumb domain, turning the conventional right-handed shape of the RT into a triangular architecture (Fig. 4a, Supplementary Figure 2). The ncRNA forms a complex series of stem loops that interact with every domain of the protein (Fig. 4b,c). The 3′ end of the ncRNA curls around the thumb, threading through the center of the triangle and forming extensive, non-base pairing interactions with the 5′ end of the ncRNA (Fig. 4b). The RT-ncRNA complex assembles into a C3 symmetric trimer (Extended Data Fig. 6g) stabilized by kissing loop interactions between SL5 (C93) of one molecule and SL5.1 (G113) of the adjacent subunit.

**Extended Data Figure 7 |:**
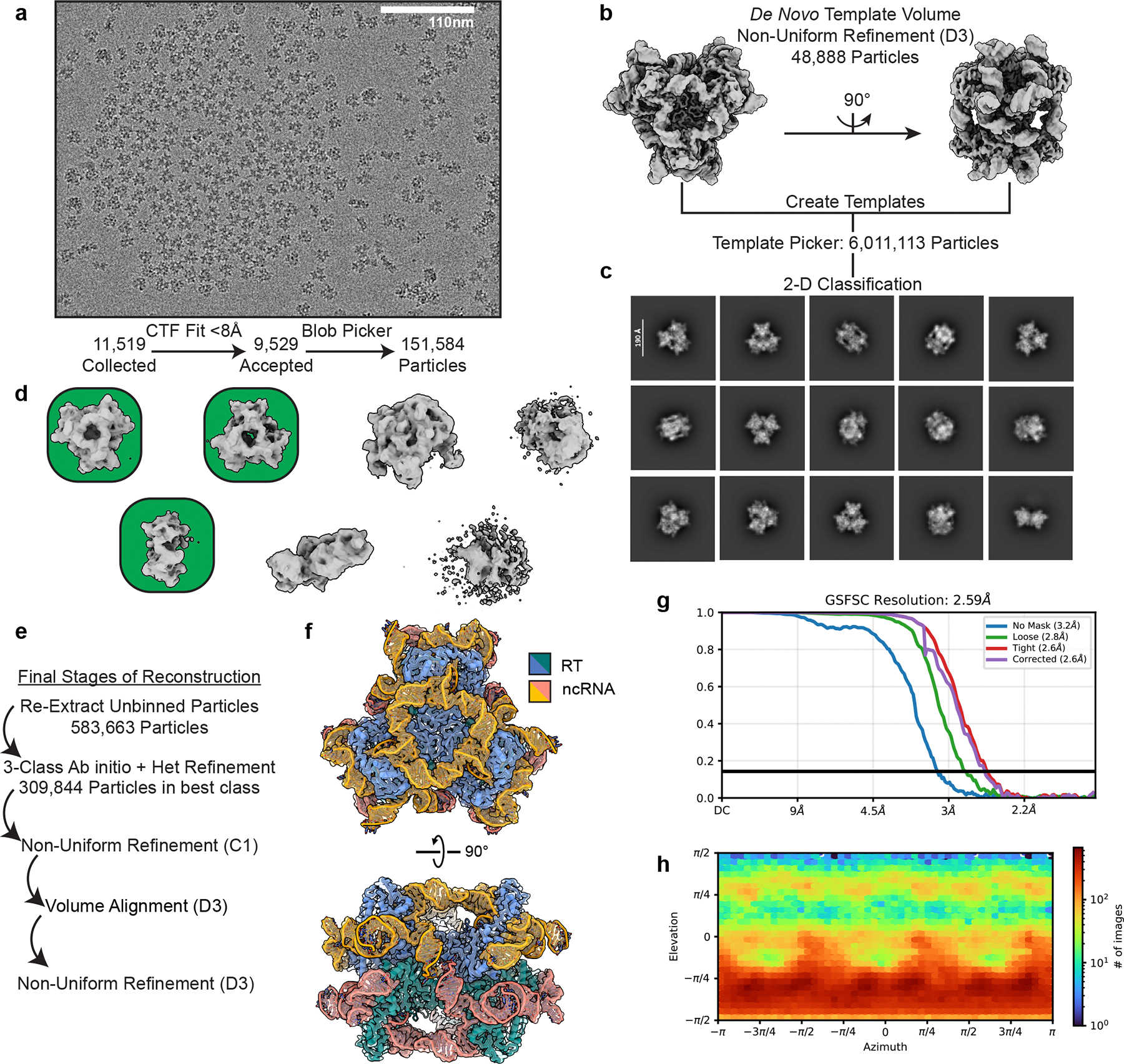
Cryo-EM analysis and image processing workflow for hexameric *Sen*DRT9 RT-ncRNA complex. **a,** Representative micrograph from the *Sen*DRT9 RT-ncRNA hexameric complex, revealing a monolayer of evenly distributed particles. **b,** Initial reconstruction from on-the-fly image analysis that was used to generate *de novo* templates used for particle picking. **c,** Representative 2D Classes from 6,011,113 template-picked particles. **d,** Initial volumes from a 7-class *ab initio* reconstruction of 1,411,369 particles. Selected volumes for downstream processing are shown with a green background. **e,** Final steps used to sort selected particles in **d** to yield the final reconstruction. **f,** Final (D3) reconstruction of the *Sen*DRT9 RT-ncRNA hexamer that refined to 2.6 Å and was used for model building. The map is shown as a partially transparent surface, with RT subunits shown in blue/cyan and ncRNA subunits shown in orange/salmon. **g,** Plot of Cryosparc’s gold standard Fourier shell correlation for the map in **f**. **h,** Plot of particle view distribution reveals mild anisotropy but no missing views.

**Extended Data Figure 8 |:**
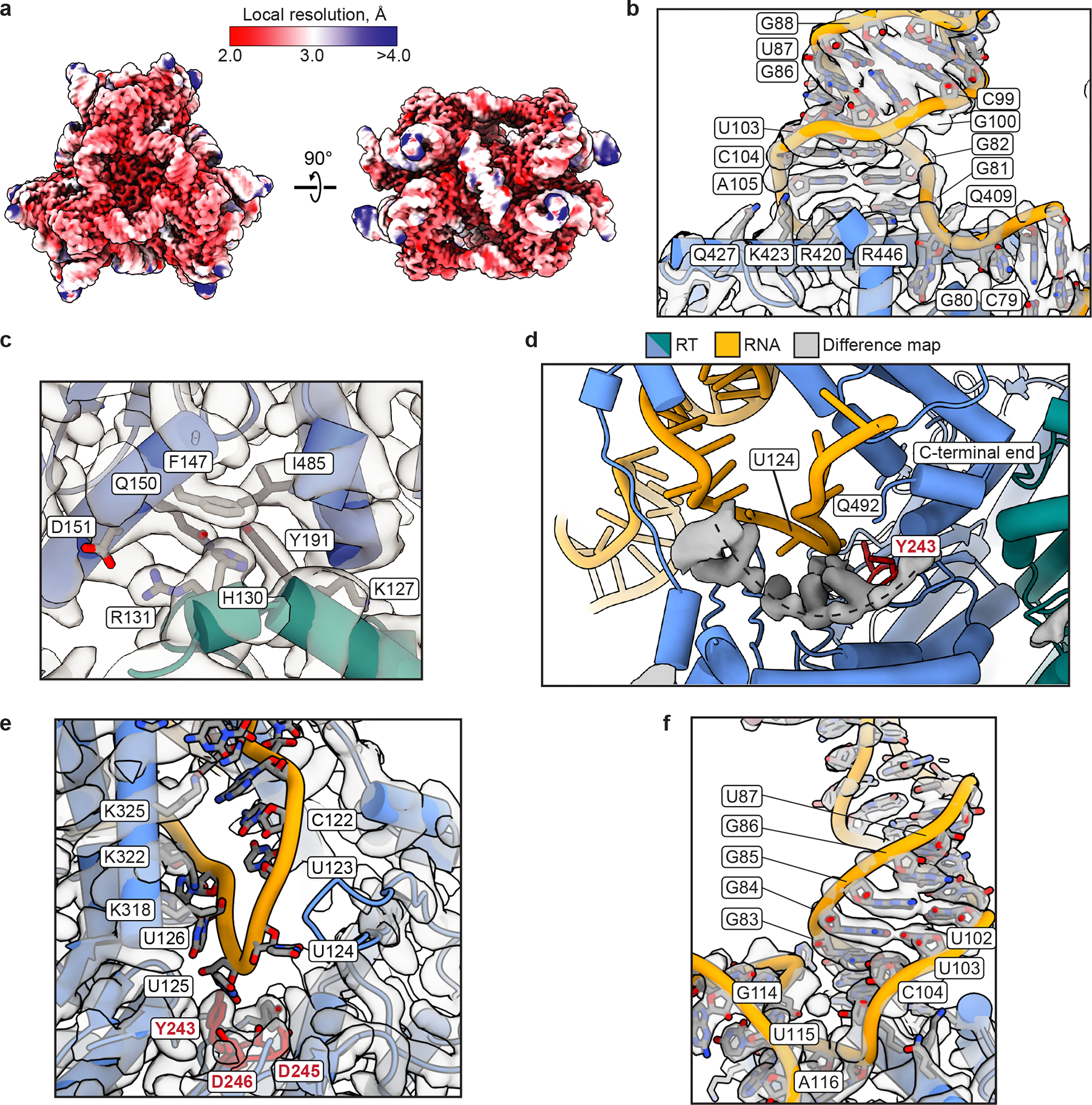
Additional features of the *Sen*DRT9 hexamer visualized by cryo-EM. **a,** Local resolution map of the *Sen*DRT9 RT-ncRNA hexamer (FSC cutoff 0.143), showing that resolution falls off near the periphery of the complex in flexible regions such as SL3 and SL4. **b,** Semi-transparent density highlighting a network of protein-RNA interactions that stabilize SL5. **c,** Semi-transparent density highlighting protein-protein interactions that stabilize the back-to-back assembly of trimers. **d,** Difference map generated by subtracting a calculated map of the model from the experimental density. Unmodelled density (grey) is visible in the map directly adjacent to the YADD active site (red), which may correspond to a small stretch of poly-dA product; repeated efforts to sort these particles failed to separate distinct conformational states. **e,** A close-up view of the *Sen*RT active site (YADD residues in red). Density for the RT is shown as a semi-transparent surface, and the density map for the ncRNA is omitted here for simplicity. **f,** Semi-transparent density map highlighting the ncRNA poly-G track (residues 80–86) that was used to establish register for ModelAngelo.

While the structure explains RT-ncRNA interactions and the role of the ncRNA in driving trimer assembly, the affinity-tagged protein is inactive for poly-dA synthesis *in vitro* and non-functional for defense against T5 phage *in vivo* (Extended Data Fig. 4d,6j). We therefore reasoned that removal of the affinity tag would likely clarify the structural basis for RT-ncRNA function. Following protease cleavage of the affinity tag, we used cryo-EM to determine a 2.6 Å-resolution reconstruction, which revealed a hexameric architecture — a back-to-back dimer of trimers (Fig. 4d, Extended Data Fig. 7,8a, Extended Data Table 1). The hexameric assembly is reminiscent of AbiK^16^, but the mechanisms of assembly are distinct (Supplementary Figure 2). Assembly of the AbiK hexamer relies on an alpha-helical repeat domain (i.e., αRep), while assembly of the DRT9 hexamer relies on a combination of RNA-RNA (SL5:SL5.1 and SL4:SL4), RNA-protein, and protein-protein interactions, resulting in a buried surface area of 3,000 Å^2^ between trimers (Fig. 4d–f, Extended Data Fig. 8b,c). Although the structures of the trimer and hexamer are nearly equivalent, the hexamer contains additional density near the active site that is not present in the trimer reconstruction (Extended Data Fig. 8d). This unmodelled density may correspond to a small stretch of poly-dA product, but repeated efforts to sort these particles failed to separate distinct conformational states. Notably, the U_4_ motif (123–126) is positioned ~8 Å from the YADD active site, consistent with a role in templating poly-dA synthesis (Fig. 4g, Extended Data Fig. 8e). Overall, these data reveal that the DRT9 RT-ncRNA complex forms a D3 symmetric hexamer that performs RNA-templated poly-dA synthesis.

## Host and viral factors modulating DRT9

**Extended Data Figure 9 |:**
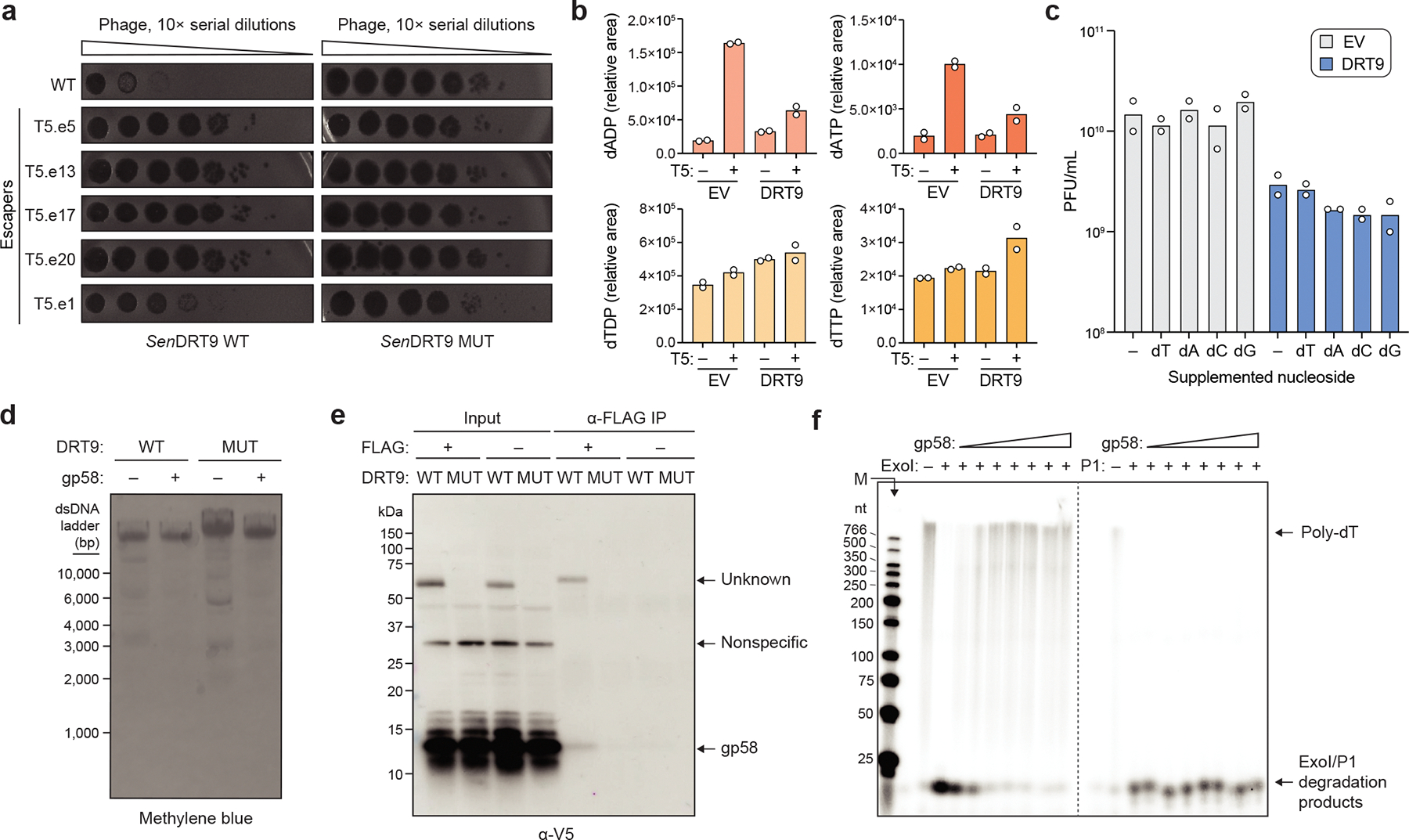
Activation of DRT9 immunity by phage-encoded factors. **a,** Representative plaque assays with WT or escaper T5 phage variants tested in strains expressing either the WT or RT-inactive (MUT) *Sen*DRT9 system. **b,** Bar graphs of relative nucleotide levels for the indicated species quantified by LC-MS/MS, in lysates from cells expressing *Sen*DRT9 or an empty vector (EV) control +/− T5 phage infection. Data are shown as the mean for n = 2 independent biological replicates. **c,** Bar graphs of plaque forming units per mL (PFU/mL) of T5 phage lysates after liquid culture infection of cells expressing *Sen*DRT9 or an EV control, in the presence of the indicated supplemented nucleosides; data are shown as the mean for n = 2 independent biological replicates. **d,** Methylene blue-stained membrane of total DNA from cells expressing WT or MUT *Sen*DRT9 +/− gp58 induction, for the Southern blot shown in Fig. 5f. **e,** Western blot analysis of *Sen*RT co-immunoprecipitation with gp58. Immunoprecipitation was performed with a FLAG antibody on lysates from cells co-expressing *Sen*RT (WT or MUT, FLAG-tagged or untagged) with V5-tagged gp58, followed by Western blot analysis using a V5 antibody. The unique presence of a band corresponding to gp58 in the IP eluate from cells expressing WT *Sen*DRT9 indicates a DNA-dependent interaction between the RT and gp58. **f,** Denaturing 5% urea-PAGE analysis of DNA polymerization assays, after reactions were treated with ExoI (left) or Nuclease P1 (right) in the presence of increasing concentrations of T5 gp58. All reactions contained 20 nM RT-ncRNA (U_4_>A_4_) and 100 μM [α-^32^P]-dTTP and were incubated at 37 °C for 60 min prior to addition of gp58 and ExoI or Nuclease P1. For **d-f,** representative data are shown for experiments repeated at least two times with similar results.

**Figure 5 |:**
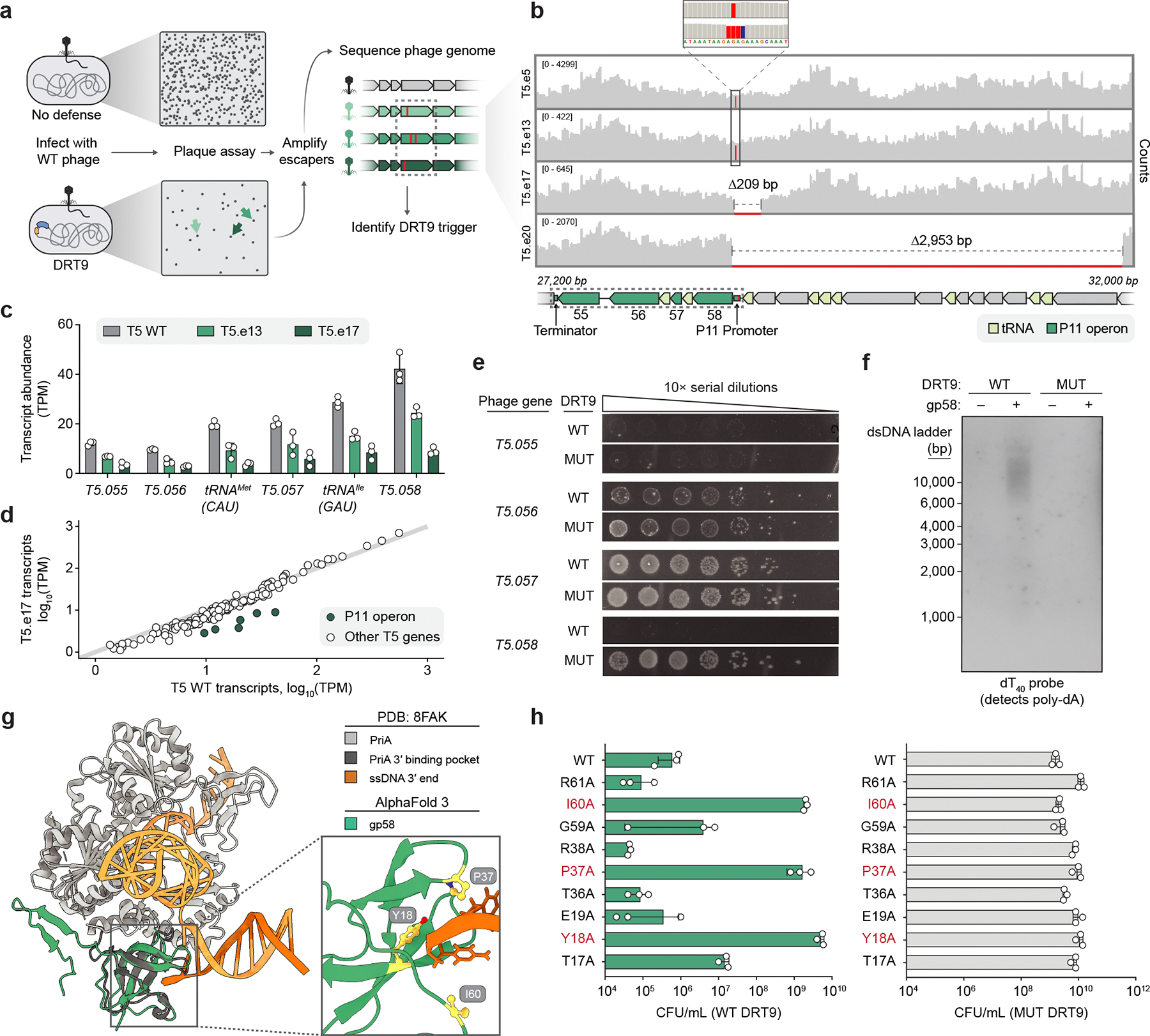
Identification of a viral trigger that activates *Sen*DRT9-mediated abortive infection. **a,** Schematic of workflow to isolate and sequence phage variants that escape detection and/or elimination by a *Sen*DRT9 immune response. **b,** Representative coverage tracks from whole-genome sequencing of T5 ‘escaper’ phage variants, shown above the corresponding genomic locus; all mutation classes shown perturb the putative P11 promoter controlling the expression of four protein-coding genes (green). **c,** Bar graph quantifying P11 operon transcript levels during infection of *Sen*DRT9-expressing cells with WT or escaper T5 phage variants, measured via RNA-seq and quantified as transcripts per million reads (TPM). **d**, Scatterplot comparing transcriptome-wide expression levels between WT T5 and T5.e17 escaper phage during infection of *Sen*DRT9-expressing cells, quantified as in **c**; P11 operon transcripts are colored in green. **e**, Colony formation assays to evaluate cell viability upon co-expression of WT or RT-inactive (MUT) *Sen*DRT9 with candidate phage genes regulated by the P11 promoter. **f,** Southern blot analysis of total DNA isolated from cells expressing WT or MUT *Sen*DRT9 +/− gp58 induction. DNA was probed with oligo-dT_40_ to detect poly-dA species; sizes from a dsDNA ladder are marked. Representative data are shown for experiments repeated at least two times with similar results. **g,** Predicted AlphaFold 3 structure of T5 gp58 (green) superimposed onto the structure of *E. coli* PriA (grey) bound to a replication fork DNA substrate (orange; PDB ID: 8FAK). The magnified inset highlights gp58 residues predicted to bind DNA 3′ ends^23^; RMSD = 0.95 Å over 47 Cα atoms. **h,** Bar graphs quantifying cell viability in colony forming units (CFU) upon co-expression of the indicated gp58 alanine substitution variants with WT (left) or MUT (right) *Sen*DRT9; mutations predicted to affect DNA 3′ end recognition are indicated with red text. Data are shown as mean ± s.d. for n = 3 technical replicates.

Our biochemical investigation into DRT9 reverse transcription activity illuminated key molecular details of poly-dA synthesis, but also introduced an apparent discrepancy: *in vitro* poly-dA synthesis occurs constitutively, whereas *in vivo* poly-dA synthesis is strictly phage-dependent. This suggested the presence of additional regulatory mechanisms that control DRT9 activation in a cellular context. To identify potential viral triggers of DRT9 activity, we screened for ‘escaper’ phages harboring mutations that confer resistance to DRT9 immunity. Using a well-established workflow to isolate escaper phages (Fig. 5a), we identified 41 independent T5 phage variants (11 unique genotypes) that evaded *Sen*DRT9, and 1 T5 phage variant and 13 independent Bas37 phage variants (4 unique genotypes) that evaded *Psa*DRT9. Phages that infected DRT9 strains similarly to an empty vector (EV) control were whole-genome sequenced to identify mutations linked to immune evasion (Extended Data Fig. 9a, Supplementary Table 3). Sequencing revealed diverse frameshift and missense mutations in *nrdA* (T5, Bas37) and *nrdB* (Bas37), which encode the large and small subunits of viral ribonucleotide reductase (RNR) complexes that catalyze ribonucleotide to deoxyribonucleotide conversion. This finding implicated nucleotide availability as an important facet of DRT9 immunity, as escaper mutations in *nrdA* and *nrdB* could limit deoxynucleotide levels in infected cells and decrease substrate availability for poly-dA synthesis. To examine how phage infection affects the nucleotide pool, either in the absence or presence of the DRT9 immune system, we performed metabolomics experiments with uninfected or T5-infected cells and found that phage T5 induced a sharp increase in adenosine nucleotide levels (Extended Data Fig. 9b). The presence of *Sen*DRT9 attenuated this increase (Extended Data Fig. 9b), consistent with the rapid consumption of dATP observed in our *in vitro* polymerization experiments (Fig. 3a). Notably, dATP levels in the context of DRT9 activation did not decrease below the levels observed in uninfected cells, arguing against the possibility that DRT9 activation might lead to abortive infection through nucleotide depletion. This was further corroborated by experiments showing that nucleoside supplementation had no discernible effect on DRT9 immunity or phage replication (Extended Data Fig. 9c), suggesting a more complex regulatory mechanism underlying DRT9 activation.

A larger set of T5 escaper mutations clustered within a well-defined genomic region and consisted primarily of deletions spanning 200–15,000 bp (Supplementary Table 3). This tRNA-rich region, known as del-1, is a hotspot for large genomic deletions due to repeat-associated recombination^20,21^. However, the discovery of additional escaper genotypes containing only intergenic point mutations within this region redirected our attention to the P11 promoter, which drives expression of a six-gene operon that includes two tRNAs (Fig. 5b). RNA-seq revealed specific downregulation of this operon during infection with escaper phages bearing P11 promoter mutations, as compared to WT T5 (Fig. 5c,d). We therefore hypothesized that one or more genes within this operon might activate the DRT9 immune response, and we cloned expression plasmids for each of the four protein-coding genes to test the effects of DRT9 and phage gene co-expression. Strikingly, a single gene, *T5.058*, prevented cell growth when co-expressed with WT *Sen*DRT9 but not the RT-inactive mutant (Fig. 5e). This was supported by Southern blotting analysis, which showed accumulation of poly-dA products in cells co-expressing *T5.058* with WT *Sen*DRT9 (Fig. 5f, Extended Data Fig. 9d), albeit at lower levels than those induced by phage infection. These results strongly implicate gp58, the *T5.058* gene product, as a necessary and sufficient T5 phage factor for triggering DRT9-mediated abortive infection.

We predicted the structure of gp58, an uncharacterized protein, and identified a high degree of structural similarity to the 3′ DNA-binding domain of PriA (Fig. 5g), a primosomal protein involved in DNA replication restart at stalled or damaged replication forks^22^. Co-immunoprecipitation experiments revealed an interaction between the RT and gp58 in cells expressing the WT *Sen*DRT9 system, but not the RT-inactive mutant (Extended Data Fig. 9e), implicating gp58 in RT- and/or poly-dA-dependent binding. To further probe the role of gp58, we used its structure prediction, alongside previous studies of DNA binding by PriA^23^, to design gp58 variants predicted to disrupt DNA interactions. Mutating gp58 in this manner restored normal growth in cells co-expressing gp58 and *Sen*DRT9, indicating failed triggering of DRT9-mediated cell death (Fig. 5h). Thus, we conclude that gp58 likely recognizes the DNA homopolymer synthesized by *Sen*DRT9 through direct binding to its 3′ end.

**Figure 6 |:**
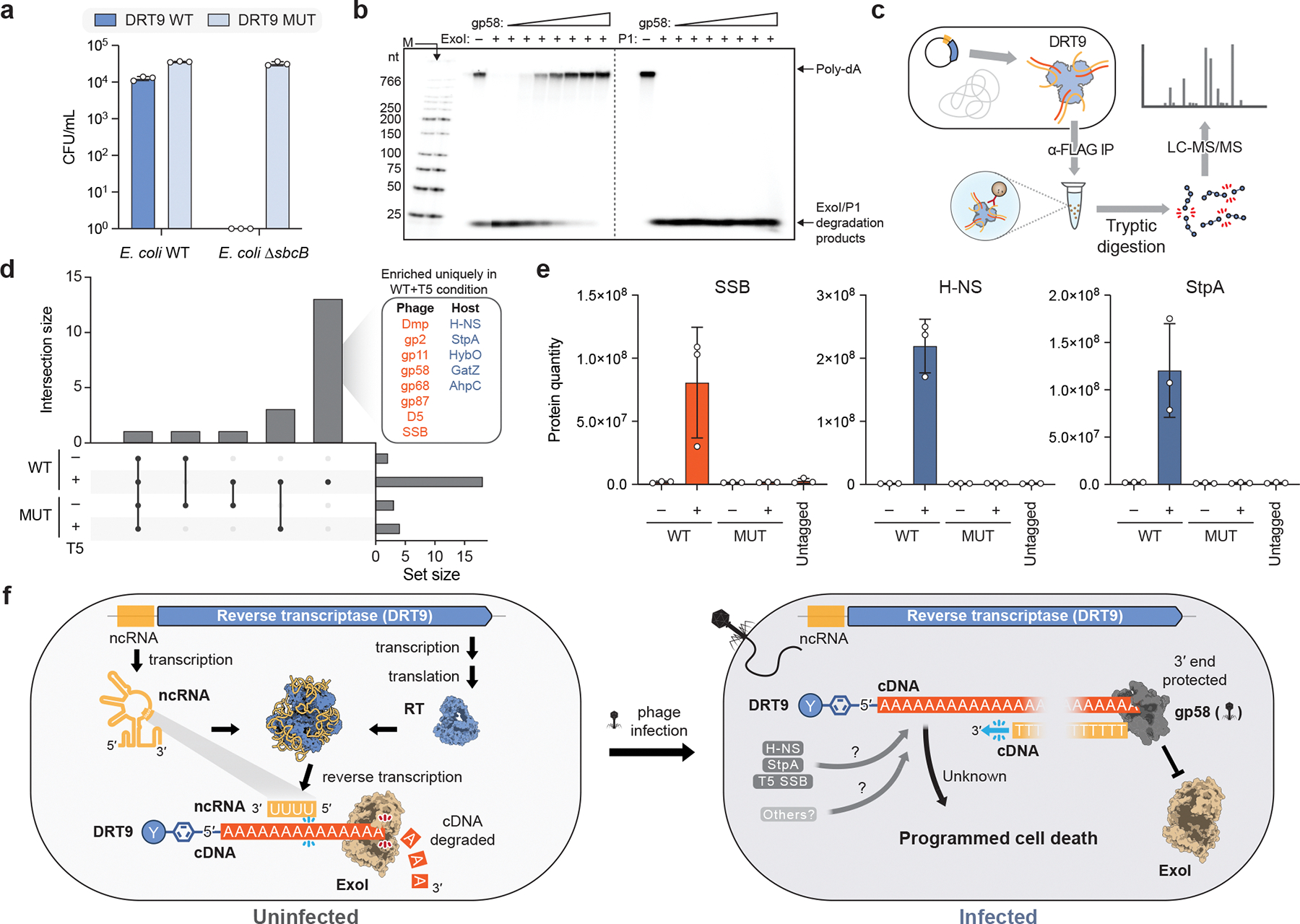
Host regulation of and response to poly-dA synthesis by DRT9 immune systems. **a,** Bar graph quantifying cell viability in colony forming units (CFU) upon transformation of WT or *ΔsbcB E. coli* with WT or RT-inactive (MUT) *Sen*DRT9, demonstrating that the *sbcB* gene product (ExoI) neutralizes the otherwise toxic properties of DRT9 synthesis products. Data are shown as mean ± s.d. for n = 3 technical replicates. **b,** Denaturing 5% urea-PAGE analysis of DNA polymerization assays, after reactions were treated with ExoI (left) or Nuclease P1 (right) in the presence of increasing concentrations of T5 gp58. Reactions were incubated at 37 °C for 10 min prior to addition of gp58 and ExoI or Nuclease P1. Representative data are shown for experiments repeated at least two times with similar results. **c,** Schematic of co-IP MS experiments to identify protein interactors of *Sen*DRT9 and its DNA synthesis products. **d,** UpSet plot showing the number of overlapping and unique protein interactors identified from co-IP MS experiments with WT or MUT *Sen*DRT9 +/− T5 phage infection; significantly enriched proteins were identified as those exhibiting >20-fold enrichment with FDR < 0.05. Phage proteins (orange) and host proteins (blue) enriched uniquely in the WT + T5 condition are labeled. **e,** Bar graph showing normalized protein intensity values for T5 SSB and *E. coli* H-NS and StpA across the indicated co-IP MS experiments from **d**, including an untagged control. Data are shown as mean ± s.d. for n = 3 independent biological replicates. **f,** Model for the antiphage defense mechanism of DRT9 systems. The RT-ncRNA hexamer constitutively synthesizes poly-dA using a U-rich template within the ncRNA. Host ExoI degrades poly-dA in uninfected cells, but phage infection leads to expression of a trigger protein (gp58 in T5) that competes for 3′ end binding, leading to poly-dA accumulation along with partial second-strand synthesis of poly-dT. Additional viral and host factors (SSB, H-NS, and StpA) bind poly-dA and/or poly-dT products, leading to programmed cell death.

Our finding that DRT9 activity is triggered by a phage protein predicted to bind DNA 3′ ends dovetailed with a separate insight that arose from literature review. A comprehensive study of *E. coli* gene essentiality previously found that *sbcB*, which encodes the 3′-to-5′ exodeoxyribonuclease I (ExoI), is an essential gene in strains expressing UG17 (an RT system related to DRT9), suggesting that ExoI mitigates the otherwise toxic effects of UG17^24^. Strikingly, we were unable to obtain transformants of a *ΔsbcB* strain with WT *Sen*DRT9, whereas the RT-inactive mutant was well-tolerated (Fig. 6a), revealing a similar conditional essentiality relationship. This discovery suggested a model in which constitutive poly-dA degradation by ExoI in uninfected cells is out-competed by poly-dA 3′ end binding by gp58 in T5-infected cells, leading to homopolymer accumulation and abortive infection. Importantly, this mechanism would resolve the apparent discrepancy between our *in vivo* observation that poly-dA synthesis occurs only after phage infection, and our *in vitro* observation that poly-dA synthesis activity occurs constitutively. In excellent agreement with this model, DRT9-derived poly-dA and poly-dT products were efficiently degraded by recombinant ExoI *in vitro*, but were protected from degradation in the presence of gp58 (Fig. 6b, Extended Data Fig. 9f). Notably, gp58 did not protect against endonuclease activity (Fig. 6b), further supporting its role in cDNA 3′ end protection.

Finally, we performed co-immunoprecipitation proteomics experiments using the FLAG-tagged RT in the absence or presence of T5 infection to identify additional viral or host factors involved in DRT9 immunity (Fig. 6c). As expected, gp58 was significantly enriched in cells expressing WT *Sen*DRT9 but not the RT-inactive mutant, corroborating our earlier findings by Western blotting. Additional T5 proteins implicated in DNA binding and genome replication were also uniquely enriched by the WT RT, including T5 single-stranded DNA binding protein (SSB) (Fig. 6d,e). Intriguingly, immunoprecipitation of the WT RT also strongly enriched two host-encoded nucleoid-associated proteins, H-NS and StpA (Fig. 6d,e). Both proteins preferentially bind AT-rich DNA regions and are key regulators of transcription and nucleoid architecture^25^, providing an elegant link between DRT9-catalyzed cDNA synthesis and associated host factors.

## Discussion

Our study reveals a mechanism of antiviral immunity mediated by DRT9 defense systems, in which a ncRNA templates the synthesis of long, kilobase-length homopolymers of deoxyadenylate to drive abortive infection (Fig. 6f). Biochemical and structural data demonstrate that a hexameric RT-ncRNA complex polymerizes dATP using a protein primer and poly-uridine template. Genetic screening revealed a phage-encoded DNA binding protein, gp58, that drives poly-dA accumulation *in vivo* by blocking its degradation by a host exonuclease, ExoI. Importantly, our finding that gp58 alone is sufficient to induce cellular toxicity in a DRT9-expressing strain argues against poly-dA synthesis acting to specifically antagonize phage infection. Instead, we propose that poly-dA accumulation drives a phage-extrinsic abortive infection mechanism.

The mechanism by which poly-dA accumulation leads to cell death remains elusive. *In vitro* reverse transcription reactions with *Sen*DRT9 rapidly consumed dATP, leading us to initially suspect that nucleotide depletion might inhibit both host and phage genome replication by starving the cell of adenosine nucleotides. If true, this mechanism would provide an elegant counterpart to other phage defense systems that similarly deplete deoxyribonucleotides^8,9^. However, while nucleotide metabolomics measurements in infected cells revealed that dATP levels were lower in the presence of DRT9, supplementation of deoxyadenosine nucleosides failed to suppress the immune response (Extended Data Fig. 9b,c). An alternative hypothesis is that poly-dA accumulation in cells leads to sequestration of specific DNA-binding proteins, as suggested by our co-immunoprecipitation experiments (Fig. 6d,e). However, previous studies of strains mutated in *hns* and/or *stpA* do not indicate that their dysfunction would be lethal to the cell^26,27^, suggesting that additional factors may be involved. A third hypothesis arises when considering that DRT9 systems sometimes associate with SLATT domain-containing proteins (Extended Data Fig. 1a), which contain transmembrane (TM) helices predicted to form toxic, membrane-embedded pores^28^. Considering recent reports of other phage defense systems that use oligonucleotides to modulate ion channel activity^29,30^, nucleic acid-mediated regulation of membrane proteins may be a broader theme in antiviral immunity. However, whether *Sen*DRT9 poly-dA products similarly engage membrane proteins in *E. coli* requires future investigation. Resolving the abortive infection mechanism of poly-dA synthesis will be a focus of future effort and will require pursuing these, and potentially additional, molecular hypotheses.

The unusual mechanism of RNA-templated homopolymer synthesis reported here sets DRT9 reverse transcriptase enzymes apart from all other known polymerases. Although nucleotidyltransferase (NTase) enzymes that polymerize RNA substrates — such as poly(A) and poly(U) polymerase — also produce nucleic acid homopolymers, they do so without a template, relying instead on protein-mediated nucleotide selection within the active site^31^. Similarly, terminal deoxynucleotidyl transferase (TdT) can add homopolymeric stretches of DNA when a given nucleotide is in excess, but lacks the specificity conferred by a nucleic acid template^32^. DRT9 enzymes are, to our knowledge, the only polymerases that harness an RNA template to direct the synthesis of DNA homopolymers, yielding kilobase-length products in a highly processive manner. Even more strikingly, these homopolymers remain covalently tethered to the enzyme itself, via a protein priming mechanism dependent on conserved tyrosine residues.

The structural architecture of DRT9 RT-ncRNA complexes is equally distinctive, especially in comparison to DRT2-family enzymes. The DRT2 RT-ncRNA complex behaves as a functional monomer^5^, whereas the DRT9 complex adopts a hexameric architecture comprising a dimer of trimers. This arrangement echoes the hexameric and trimeric assemblies of two other UG-family reverse transcriptases, AbiK and Abi-P2^16^, but is uniquely stabilized by RNA-mediated interactions that form a tightly interlinked cage to encapsulate the enzyme subunits. The proximity of the U-rich template region to the catalytic pocket provides a structural framework for understanding the mechanism of poly-dA synthesis. Indeed, the proposed mechanism by which DRT9 RTs iteratively engage the same template region for repetitive DNA synthesis is highly reminiscent of repeat addition processivity, a hallmark feature of telomerase reverse transcription activity^33^. Future studies will be needed to determine the precise number of uridines required for templated deoxyadenosine incorporation, how the nascent cDNA is realigned within the active site and, importantly, how kilobase-sized homopolymers extrude from the body of the hexameric complex while remaining covalently tethered at the 5′ end and engaged within the active site at the 3′ end.

As with DRT2, insights into DRT9 arose from questioning conventional assumptions about RT enzymes and their cDNA synthesis products. In both cases, a departure from standard approaches in high-throughput sequencing data analysis allowed us to identify unexpected cDNA products. Notably, our initial interest in DRT9 stemmed from the hypothesis that its properties would closely resemble DRT2, given their close phylogenetic relationship, but our findings here illuminate a dramatically different mechanism of antiviral immunity. As such, we anticipate that further exploration of reverse transcriptase diversity will continue to uncover unexpected DNA synthesis mechanisms, and that the full scope of nucleic acid functions in antiviral defense is only beginning to come into view.

## METHODS

### Plasmid and *E. coli* strain construction.

All strains and plasmids used in this study are described in Supplementary Tables 4–5, respectively. DRT9 operons, including their native upstream and downstream flanking sequences, were chemically synthesized (Twist Bioscience or GenScript) and cloned into a pACYC184 plasmid backbone by Gibson Assembly. Derivative plasmids were cloned using a variety of methods, including around-the-horn PCR, restriction digestion-ligation, and Golden Gate Assembly. All plasmids were cloned and propagated in *E. coli* strain NEB Turbo (sSL0410). Clones were verified by Sanger sequencing or whole plasmid sequencing. Substitution and insertion mutations to the *Sen*DRT9 ncRNA are numbered relative to the first nucleotide of the ncRNA, with insertion numbering referencing the nucleotide immediately upstream of the inserted bases (for instance, A109U indicates mutation of A at position 109 to U, and 124insU indicates insertion of U downstream of position 124). Experiments were performed in *E. coli* K-12 strain MG1655 unless otherwise indicated. The Δ*sbcB* strain from the Keio collection^34^, which harbors a single-gene deletion in *E. coli* K-12 strain BW25113, was a gift from S. Tavazoie.

### Phylogenetic analyses.

The phylogenetic tree of UG RT homologs in Fig. 1a was visualized using data from Mestre *et al*.^3^. For visualization, each UG/DRT clade was collapsed into a single representative, using the longest branch within each clade. To build the phylogenetic tree of DRT9 RT homologs in Extended Data Fig. 1a, a BLASTp search of a local copy of the NCBI NR database (downloaded April 4, 2023) was queried with 124 DRT9 (i.e., UG28) sequences identified by Mestre et al.^3^ (-evalue 0.01 -max_target_seqs 1,000,000), resulting in the identification of 438 unique DRT9 homologs. These proteins were aligned with MAFFT^35^ (LINSI option) and a phylogenetic tree was constructed from the resulting alignment with FastTree [-wag -gamma options]^36^. Genomes encoding these DRT9 homologs were then downloaded using NCBI’s Batch-Entrez function, and loci comprising *drt9* plus 10 kbp of upstream and downstream nucleotide sequence were extracted. Next, to identify DRT9 systems that encode a SLATT domain-containing gene, we predicted ORFs in each DRT9 locus using Eggnogg Mapper^37^ and used HMMER^38^ to search the resulting ORFs with a previously built hidden Markov model (PFAM: PF18160). Finally, ncRNA associations were annotated using the CMsearch function of Infernal^39^ to search DRT9 loci with a CM built from DRT9-associated ncRNAs described below.

### Sequence identity matrices.

Sequence identity between selected DRT9 homologs was analyzed by performing MAFFT alignment of RT amino acid sequences using default settings. Accession numbers for RT proteins are listed in Supplementary Table 2.

### Phage propagation and plaque assays.

Phages T2, T5, and λ-vir were gifts from Michael Laub. BASEL collection phages were gifts from Alexander Harms. All phages were propagated in liquid culture by picking single plaques into LB media, supplemented with 5 mM CaCl_2_ and 5 mM MgSO_4_, containing *E. coli* MG1655 cells diluted 1:100 from overnight cultures. After 4–5 hours of incubation with shaking at 37 °C, chloroform was added to a final concentration of 5% to lyse residual bacteria, and lysates were centrifuged at 4,000 x *g* for 10 min to pellet cell debris. Supernatants were passed through a sterile 0.22 μm filter and stored at 4 °C.

Small-drop plaque assays were performed as previously described^4^. Briefly, *E. coli* K-12 strain MG1655 (sSL0810) (see Supplementary Table 4 for strain descriptions and genotypes) was transformed with the indicated plasmids (see Supplementary Table 5 for plasmid descriptions and sequences). Transformants were inoculated in liquid LB media containing the appropriate antibiotic and grown overnight at 37 °C with shaking. The next day, overnight cultures were mixed with molten soft agar (0.5% agar in LB media supplemented with 5 mM CaCl_2_ and 5 mM MgSO_4_) at 45 °C and poured over solid bottom agar (1.5% agar in LB media containing the appropriate antibiotic) in a Petri dish. 10× serial dilutions of phage in LB media were spotted onto the surface of the soft agar lawn. Plates were incubated at 37 °C for 8–16 hours to allow plaque formation. Plaque forming units (PFU) mL^−1^ were calculated using the following formula: $\frac{number\;of\;plaques}{0.003\;mL\;x\;dilution\;factor}$. When individual plaques were indistinguishable, a count of 50 plaques was assigned at the lowest dilution showing visible lawn clearance. Phage defense activity was assessed by calculating the fold reduction in efficiency of plating (EOP), which was determined by dividing the PFU mL^−1^ obtained on a lawn of empty vector (EV)-transformed control cells by the PFU mL^−1^ obtained on a lawn of defense system-expressing cells.

### Plaque and colony formation time course.

Phage replication and cell viability over the course of T5 infection were measured as previously described^4^. *E. coli* MG1655 cells were transformed with plasmids encoding WT or MUT *Sen*DRT9 and grown to OD_600_ of 0.4 in LB media supplemented with chloramphenicol (25 μg/mL). For the t = 0 time point, an aliquot of T5 phage lysate was taken for plaque counting, and an aliquot of uninfected culture was taken for colony counting. Infections were initiated by adding phage at an MOI of 5. The cultures were incubated with shaking at 37°C for 90 min, and aliquots of each infection were taken every 30 min for plaque and colony counting.

For plaque forming unit (PFU) measurements, 100 μL of each culture was taken at the indicated time point and mixed with chloroform (4% final concentration) to lyse cells and terminate infections. Lysates were centrifuged at 4,000 × *g* for 3 min to pellet cell debris. Supernatants containing phages were serially diluted in LB, and PFU/mL were measured using the plaque assay protocol described above.

For colony forming unit (CFU) measurements, 200 μL of each culture was collected at the indicated time point and centrifuged at 4,000 x *g* for 3 min. Pellets were washed in 1 mL LB and centrifuged again to remove residual phage particles, and then resuspended in fresh LB. Serial dilutions of cells were prepared and 5 μL of each dilution was spotted on an LB-agar plate supplemented with chloramphenicol (25 μg/mL). The plates were incubated overnight at 37 °C and colonies were counted the next day. CFU/mL were calculated using the following formula: $\frac{number\;ofcolonies}{0.005\;mL\;x\;dilution\;factor}$.

### AlphaFold structural modeling.

Protein sequences were submitted to the AlphaFold 3 webserver^40^ and the resulting structural predictions were rendered with ChimeraX^41^.

### RNA and cDNA immunoprecipitation and sequencing (RIP-seq and cDIP-seq).

RIP-seq and cDIP-seq were performed as previously described^4^. *E. coli* K-12 strain MG1655 (sSL0810) cells were transformed with plasmids encoding N-terminally 3xFLAG-tagged DRT9 systems with a WT or mutant (YAAA) RT active site (see Supplementary Table 5 for plasmid sequences). Individual colonies for each replicate experiment were inoculated in 40 mL liquid LB and grown at 37 °C to OD_600_ of 0.3–0.4. For experiments performed in the absence or presence of phage infection, cultures were split in half and phage T5 was added to one half at a multiplicity of infection (MOI) of 5. Cultures were further incubated at 37 °C with shaking for 45 min before harvesting. For experiments performed only in uninfected cells, 20 mL cultures were grown to OD_600_ of 0.5 and harvested. Cells were collected by centrifugation at 4,000 x *g* for 10 min at 4 °C and then washed with 5 mL of cold TBS (20 mM Tris-HCl pH 7.5 at 25 °C, 150 mM NaCl). Cells were centrifuged again at 4,000 x *g* for 5 min at 4 °C. Pellets were washed with 1 mL of cold TBS and centrifuged at 10,000 x *g* for 5 min at 4 °C. After supernatants were removed, pellets were flash-frozen in liquid nitrogen and stored at −80 °C.

To prepare antibody–bead complexes for immunoprecipitation, Dynabeads Protein G (Thermo Fisher Scientific) were washed 3× in 1 mL IP Lysis Buffer (20 mM Tris-HCl pH 7.5 at 25 °C, 150 mM KCl, 1 mM MgCl_2_, 0.2% Triton X-100), resuspended in 1 mL IP Lysis Buffer, combined with anti-FLAG antibody (Sigma-Aldrich, F3165), and rotated for > 3 hours at 4 °C. 60 μL of beads and 20 μL of antibody mix were prepared per sample. Antibody-bead complexes were washed 2× to remove unbound antibodies and resuspended in IP Lysis Buffer to a final volume of 60 μL per sample.

Flash-frozen pellets were thawed on ice and resuspended in 1.2 mL IP Lysis Buffer supplemented with 1× Complete Protease Inhibitor Cocktail (Roche) and 0.1 U μL−1 SUPERase•In RNase Inhibitor (Thermo Fisher Scientific). Cells were lysed by sonication and lysates were cleared by centrifugation at 21,000 x *g* for 15 min at 4 °C. Supernatants were transferred to new tubes and two 10 μL aliquots of each sample (one for RIP-seq and one for cDIP-seq) were set aside and stored at −80 °C as “input” controls. The remainder of each sample was combined with 60 μL of antibody-bead complex and rotated overnight at 4 °C. The next day, each sample was washed 3× with 1 mL ice-cold IP Wash Buffer (20 mM Tris-HCl pH 7.5 at 25 °C, 150 mM KCl, 1 mM MgCl_2_). During the final wash, each sample was split into two 500 μL volumes for downstream RIP or cDIP processing.

RIP elution was performed by removing supernatants and resuspending beads in 750 μL TRIzol (Thermo Fisher Scientific). After 5 min incubation at RT, supernatants containing eluted RNA were transferred to new tubes. Samples were then mixed with 150 μL chloroform, incubated at RT for 2 min, and centrifuged at 12,000 x *g* for 15 min at 4 °C. RNA was isolated from the upper aqueous phase using the RNA Clean & Concentrator-5 kit (Zymo Research). RNA from input samples was isolated in the same manner using TRIzol and column purification. cDIP elution was performed by removing supernatants, resuspending beads in 90 μL IP Wash Buffer, and treating samples with 5 μg RNase A (Thermo Fisher Scientific) and 25 μg Proteinase K (Thermo Fisher Scientific). Supernatants containing eluted DNA were transferred to new tubes. DNA was isolated using the Monarch Spin PCR and DNA Cleanup kit (NEB), following the Oligonucleotide Cleanup protocol. Input samples were also treated with RNase A and Proteinase K prior to DNA isolation by column purification.

For RIP-seq library preparation, RNA was diluted in NEBuffer 2 and heated at 92 °C for 2 min for fragmentation by alkaline hydrolysis. Samples were then treated with TURBO DNase (Thermo Fisher Scientific), RppH (NEB), and T4 PNK (NEB) to remove DNA and prepare RNA ends for adapter ligation. RNA was purified using the Zymo RNA Clean & Concentrator-5 kit. Adapter ligation, reverse transcription, and indexing PCR were performed using the NEBNext Small RNA Library Prep kit. Libraries were sequenced on an Element AVITI in paired-end mode with 75 or 150 cycles per end.

For cDIP-seq library preparation, samples were heated at 95 °C for 2 min and then immediately placed on ice to denature DNA. Adapter ligation and conversion of ssDNA to dsDNA were performed using the xGen ssDNA & Low-Input DNA Library Prep Kit (IDT). Indexing PCR was performed using NEBNext Ultra II Q5 Master Mix. Libraries were sequenced on an Element AVITI in paired-end mode with 75 or 150 cycles per end.

### RIP-seq analysis.

RIP-seq datasets were processed as previously described^4^. Cutadapt^42^ (v4.2) was used to remove adapter sequences, trim low-quality ends from reads, and filter out reads shorter than 15 bp. Reads were mapped to reference files containing the MG1655 genome (NC_000913.3), relevant plasmid sequence, and T5 (NC_005859.1) genome, using bwa-mem2^43^ (v2.2.1) with default parameters. SAMtools^44^ (v1.17) was used to sort and index alignments. Coverage tracks for top and bottom strand alignments were generated using bamCoverage^45^ (v3.5.1) with normalization for sequencing depth (based on the total number of reads passing initial trimming and length filtering). Coverage tracks were visualized in IGV^46^.

For transcriptome-wide analysis of RNAs enriched by RIP-seq, aligned reads were assigned to annotated transcriptome features using featureCounts^47^ (v2.0.2) with -s 1 for strandedness. Counts matrices were analyzed using DESeq2^48^ to calculate the enrichment (fold-change) and false discovery rate for each transcript compared between input and IP samples. Transcriptome-wide comparisons were visualized as MA plots, using ggplot2 to plot “baseMean” (mean normalized counts across all conditions) against log_2_(enrichment). All comparisons included three independent biological replicates.

### Total RNA sequencing.

*E. coli* MG1655 cells expressing wild-type *Sen*DRT9 were grown in liquid culture until OD_600_ reached 0.4. Cells were infected with WT phage T5 or the indicated escaper phage (T5.e13 or T5.e17, see Supplementary Table 3) at MOI = 5. Samples were collected after 10 minutes of infection by centrifuging 1 mL of culture at 4,000 x *g* for 3 min, removing the supernatant, and freezing the pellet in liquid nitrogen. To extract RNA, the pellet was resuspended in 750 μL of TRIzol, incubated at RT for 5 min and mixed with 150 μL chloroform. After incubation at RT for 2 min, samples were centrifuged at 12,000 x *g* for 15 min at 4 °C. RNA was isolated from the upper aqueous phase using the RNA Clean & Concentrator-5 kit (Zymo Research). Library preparation, sequencing, and read processing was performed as described above for RIP-seq. After removing low-abundance transcripts (mean counts < 10), gene expression was quantified by dividing counts by gene length and scaling to one million total counts per sample, resulting in transcripts per million (TPM) expression values.

### cDIP-seq analysis.

Adapter trimming, quality trimming, and length filtering of cDIP-seq and corresponding input (total DNA) datasets were performed as described above for RIP-seq experiments. Trimmed and filtered reads were mapped to combined reference files, sorted, indexed, and plotted onto coverage tracks as described above. For transcriptome-wide analysis of cDIP-seq data, alignments over annotated transcriptome features were counted using featureCounts with -s 2 for strandedness. Counts matrices were processed by DESeq2 and plotted as described above. The *lacZ* and *racR* loci were masked from this analysis, as they were previously found to show artifactual enrichment by cDIP-seq^4^. All transcriptome-wide comparisons were performed using three independent biological replicates.

### Unmapped read analysis.

For analysis of unmapped reads in cDIP-seq and total RNA-seq datasets (using the input controls from RIP-seq experiments), reads were first stringently trimmed and filtered to remove all residual adapter sequences. Cutadapt was used to process reads, adapting a previously described method for tiling across the 3′ adapter^49^, with error, overlap, quality, and length thresholds set at -e 0.2 -O 10 -q 30 -m 45. The fraction of unmapped reads for each sample was calculated using SAMtools flagstat. Unmapped reads were then extracted for downstream analysis using SAMtools fasta. Motif analysis was performed using MEME^50^ (v5.5.7) in differential enrichment mode with minimum width 45 (-minw 45) and allowing any number of motif repetitions (-mod anr), using unmapped reads from cDIP-seq of the catalytically inactive RT (YAAA) as the control sequence set. Quantification of homopolymer-containing reads was performed using countPattern from the Biostrings package (v2.70.3) in R. Reads were analyzed for the presence of at least 25 consecutive A, T, G, or C bases, or for the presence of A_10_T_10_ or T_10_A_10_ sequences, allowing 1 mismatch. Reads containing homopolymers were counted and normalized according to the total number of filtered reads for each sample.

### Oligonucleotide spike-in sequencing.

To assess potential biases in library preparation and sequencing of poly-dA and poly-dT, 100-nt single-stranded DNA homopolymers (oligo-dA and oligo-dT) were chemically synthesized (IDT) and analyzed by next-generation sequencing. 250 pmol oligo-dA and oligo-dT were each 5′-phosphorylated by treatment with T4 PNK (NEB) for 30 min at 37 °C. Reactions were cleaned up using the Monarch Spin PCR and DNA Cleanup Kit (NEB) and eluted in 20 μL water. Phosphorylated oligos were added to ~5 pmol total DNA — extracted from *E. coli* K-12 strain MG1655 cells expressing catalytically inactive *Sen*DRT9 — in various amounts (50 fmol oligo-dA only, 50 fmol oligo-dT only, 50 fmol each of oligo-dA and oligo-dT, or 500 fmol of oligo-dA and 50 fmol of oligo-dT). Spiked-in DNA samples were subjected to the same library preparation and sequencing workflow as described above for cDIP-seq.

### Southern blotting.

DNA samples were collected from either uninfected or phage T5 infected cells. Cultures from a single colony were grown in 20 mL liquid culture until OD_600_ reached 0.3. For experiments performed in presence of phage infection, phage T5 was added at a multiplicity of infection (MOI) of 5. For uninfected cells, an equal volume of LB was added. Cultures were further incubated at 37 °C with shaking for 60 min before harvesting. For experiments containing the gp58 trigger, cells were grown in 20 ml LB supplemented with chloramphenicol (25 μg/mL), spectinomycin (100 μg/mL) and 2% glucose until they reached OD_600_ of 0.3. Cells were pelleted by centrifugation at 4,000 x *g* for 5 min, washed with 10 mL LB and resuspended in 20 mL LB with chloramphenicol (25 μg/mL), spectinomycin (100 μg/mL) and 0.2% L-arabinose to induce gp58 expression. Cultures were incubated at 37 °C with shaking for 45 min before harvesting. Cells were collected by centrifugation at 4,000 x *g* for 10 min at 4 °C and then washed with 5 mL of cold TBS. Cells were centrifuged again at 4,000 x *g* for 5 min at 4 °C. Pellets were washed with 1 mL of cold TBS and centrifuged at 10,000 x *g* for 5 min at 4 °C. Afterwards supernatants were removed and pellets were stored at −20 °C.

Genomic DNA samples were extracted using the Wizard Genomic DNA Purification Kit (Promega). 3 μg of gDNA were loaded on an agarose gel (0.8% agarose, 1× TAE Buffer) and run for 2 hours at 90 V. The gel was soaked for 10 min in Denaturation Buffer (0.5 M NaOh, 1.5 M NaCl), and DNA was transferred to a Hybond-N+ membrane (GE Healthcare) by upward capillary transfer in Alkaline Transfer Buffer (0.25 M NaOH, 1.5 M NaCl). The next day, DNA was denatured for probe hybridization by incubating the membrane in 0.4 M NaOH for 10 min, then rinsed in Na_2_HPO_4_ (0.5 M, pH 7.3) and DI water. DNA transfer was confirmed by staining the membrane with methylene blue and imaging with an Amersham Imager 600 (GE Healthcare). The membrane was pre-hybridized in ULTRAhyb-Oligo Buffer (Thermo Fisher Scientific) for 1 hour at 42 °C. A biotinylated oligonucleotide probe of poly-dT_40_ or poly-dA_40_ was added to the Hybridization Buffer at a final concentration of 5 nM and hybridization was performed overnight at 42 °C. The next day, the membrane was washed twice with Southern Blot Wash Buffer I (2× SSC with 0.1% SDS) and twice with Southern Blot Wash Buffer II (0.1× SSC with 0.1% SDS). The membrane was developed using the Chemiluminescent Nucleic Acid Detection Module Kit (Thermo Fisher Scientific) and imaged with an Amersham Imager 600 (GE Healthcare). Probe sequences are provided in Supplementary Table 6. Uncropped gel and blot images are provided in Supplementary Figure 1.

For the samples subjected to proteinase K treatment, 3 μg of gDNA were incubated with 1 μL of Proteinase K (NEB) in 1× rCutsmart buffer (NEB) at 55 °C for 30 min. Enzyme inactivation was performed by heating the reaction to 95 °C for 10 min. Reactions were mixed with 6× Purple Gel Loading Dye (NEB) and loaded on an agarose gel. Southern blotting was performed as described above.

### ncRNA covariance modeling.

Related homologs of DRT9 were identified using the amino acid sequence of a *Salmonella enterica* homolog (*Sen*DRT9, EAO1508900.1) as the seed query in a BLASTp search on the NR protein database (max target sequences = 100). Nucleotide sequences 1 kb upstream and downstream of RT genes were retrieved, clustered at 99.9% sequence identity to remove replicates using CD-HIT^51^ (v4.8.1), and aligned using MAFFT^52^ (v7.505). The resulting alignment was trimmed at the 5′ and 3′ ends to the precise boundaries of the ncRNA as determined by RIP-seq on *Sen*DRT9. These putative ncRNA sequences were clustered at 95% sequence identity using CD-HIT and realigned using mLocARNA^53^ (v1.9.1) with default parameters. The resulting structure-based multiple alignment was used to build and calibrate a covariance model (CM) using the Infernal suite^39^ (v1.1.4). The CMsearch function of Infernal was then used to scan through nucleotide sequences of RT and 1-kb flanking windows, generated by an expanded BLASTp search (max target sequences = 5000) seeded on *Sen*DRT9 and clustered at 85% sequence identity using CD-HIT. The final CM was evaluated for statistically significant co-varying base pairs with R-scape^54^ at an E-value threshold of 0.05, and incorporated a total of 201 homologous DRT9 systems, including *Sen*DRT9.

### ncRNA polyuridine conservation analysis.

A window encompassing the polyuridine tracks was extracted from the alignment of DRT9-associated ncRNAs described above, using the *Sen*DRT9 ncRNA as a reference. Next, columns composed of more than 50% gaps were removed with Trimal^55^. A weblogo was then generated from this alignment, before uridine occupancies were calculated for each position with the Biostrings package (v2.74.0) in R and plotted with ggplot2 (v3.5.1).

### *Sen*DRT9 RT-ncRNA complex purification.

To purify the His_6_-GST-tagged RT in complex with its ncRNA, *E. coli* BL21-AI cells (NEB) were transformed with the expression vector and cells were grown in LB broth at 37 °C to an OD_600_ of 0.6. Cultures were induced with 0.2 mM IPTG and 0.1% arabinose for overnight expression at 16 °C. Cells were pelleted by centrifugation at 3000 x *g* for 25 min at 4 °C, prior to lysis by sonication in Lysis Buffer I (20 mM Tris-HCl, pH 8.0, 150 mM NaCl, 5 mM MgCl_2_, 5% glycerol, 10 mM imidazole, 0.1% Triton-X-100, 1 mM TCEP). The lysate was clarified by centrifugation at 10,000 x *g* for 30 min at 4 °C. Clarified lysates were passed through a pre-equilibrated Ni-NTA column (Cytiva) using an AKTA purification system (GE Healthcare), then rinsed with 10 column volumes of Wash Buffer (20 mM Tris-HCl, pH 8.0, 150 mM NaCl, 5 mM MgCl_2_, 5% glycerol, 20 mM Imidazole, 1 mM TCEP) prior to elution with Lysis Buffer I supplemented with 300 mM imidazole. The collected fractions were combined and concentrated (100K MWCO PES Spin-X UF concentrator, Corning), prior to size exclusion chromatography using a Sup200 16/600 gel filtration column (Cytiva) pre-equilibrated in Protein Storage Buffer (20 mM Tris-HCl, pH 8.0, 150 mM NaCl, 5 mM MgCl_2_, 5% glycerol, 1 mM TCEP). Fractions containing the complex were pooled and treated with TEV protease overnight before another separation in the same gel filtration column.

Purification of His_6_-2XStrep-SUMO-tagged RT in complex with its ncRNA followed the same culture and induction steps. Supernatants collected after cell lysis were passed through a Strep-Tactin column (IBA) pre-equilibrated in Lysis Buffer II (20 mM Tris-HCl, pH 8.0, 150 mM NaCl, 5 mM MgCl_2_, 5% glycerol, 1 mM TCEP) and eluted with 2.5 mM desthiobiotin (IBA) in Lysis Buffer II. Pooled fractions were concentrated and further purified using a Superose 6 10/300 column (Cytiva). Fractions containing the complex were pooled and treated with Ulp1 protease overnight, before another separation by the same column. The final fractions were pooled, concentrated, aliquoted, flash frozen in liquid nitrogen, and stored at −80 °C.

To purify the dATP homopolymer associated *Sen*DRT9-ncRNA complex, we first incubated 150 nM *Sen*DRT9-ncRNA with 0.9 mM dATP for 30 min at 37 °C. The mixture was analyzed using a Superose 6 10/300 (Cytiva), equilibrated in Protein Storage Buffer (20 mM Tris-HCl, pH 8.0, 150 mM NaCl, 5 mM MgCl_2_, 5% glycerol, 1 mM TCEP).

### Analysis and sequencing of ncRNA co-purifying with RT.

Nucleic acids co-purifying with *Sen*DRT9-encoded RT were extracted by treating the RT-ncRNA complex with buffer-saturated phenol (pH 8.5). After vortexing, the sample was centrifuged at 12,000 × *g* for 4 mins, after which the aqueous phase was transferred to a fresh tube and mixed with chloroform. Following an additional round of centrifugation, the recovered aqueous phase was clarified using the Monarch RNA Cleanup Kit (NEB). For analytical RNase/DNase treatments, the purified ncRNA (200 ng) was treated with either 100 Units/mL TurboDNase (Invitrogen) or 250 Units/mL RNase A/T1 (Thermo Scientific) in a 10 μL reaction and incubated for 10 min at 37 °C. Reactions were quenched using 2× RNA Loading Dye (NEB) and heated at 70 °C for 10 min, before electrophoretic separation on a denaturing 10% urea-PAGE gel. Gels were stained with SYBR Gold. RNA-seq library preparation was performed as described above for RIP-seq samples. In brief, RNA was diluted in NEBuffer 2 and heated to 92 °C for 2 min for fragmentation by alkaline hydrolysis, prior to treatment with TURBO DNase (Thermo Fisher Scientific), RppH (NEB), and T4 PNK (NEB). Adapter ligation, reverse transcription, and indexing PCR were performed using the NEBNext Small RNA Library Prep kit. Libraries were sequenced on an Element AVITI in paired-end mode with 150 cycles per end.

### T5 phage gp58 purification.

T5 phage-encoded gp58 was purified similarly to the *Sen*DRT9 RT-ncRNA complex, with the following exceptions. Gp58 was expressed as an N-terminal His_6_-2XStrep-SUMO fusion, and was over-expressed in *E. coli* BL21-AI cells (NEB). Cells were induced at OD_600_ of 0.5 with 0.3 mM IPTG and 0.1% arabinose. After overnight induction at 16 °C, cells were centrifuged at 3000 x *g* for 25 min at 4 °C, sonicated in Lysis Buffer III (20 mM Tris-HCl, pH 8.0, 300 mM NaCl, 5% glycerol), and subjected to affinity chromatography using Strep-Tactin column (IBA). Elution was done in a Lysis Buffer III supplemented with 250 mM desthiobiotin (IBA). The fractions were pooled together, treated with Ulp1 overnight, and passed through a Ni-NTA column equilibrated in the Lysis Buffer III. The flowthrough was collected, concentrated, and was further purified by size exclusion chromatography using a Superdex 75 column (Cytiva) pre-equilibrated in gp58 Storage Buffer (20 mM Tris-HCl, pH 8.0, 300 mM NaCl, 5% glycerol). Fractions were pooled, concentrated, aliquoted, flash frozen in liquid nitrogen, and stored at −80 °C.

### Biochemical poly-dA synthesis assays.

Reverse transcription reactions generally contained *Sen*DRT9-encoded RT-ncRNA complexes and nucleotide substrates in Polymerization Buffer I (50 mM Tris-HCl, pH 8.5, 100 mM NaCl, 2 mM MgCl_2_, 5 mM TCEP), at concentrations indicated in associated figure legends. Reactions were initiated by the addition of dATP, typically present at a concentration of 100 μM, and were incubated at 37 °C for 10 min, unless otherwise stated. Radioactive experiments contained trace [α-^32^P]-dATP together with unlabeled (cold) dATP. Reactions were quenched and treated with various nuclease or proteinase reagents prior to electrophoretic separation, as indicated in figure legends. Experiments involving Nuclease P1 digestion were instead incubated in Polymerization Buffer II (50 mM Tris-HCl, pH 7.0, 100 mM NaCl, 2 mM MgCl_2_, 5 mM TCEP) to achieve more optimal conditions for Nuclease P1 cleavage, which loses activity at alkaline pH.

Reactions investigating the fate of unlabeled or radiolabeled [α-^32^P]-dATP substrates were analyzed by denaturing urea-PAGE at 5–10% acrylamide concentrations. Samples were mixed in equal volumes with 2× RNA Loading Dye (NEB) prior to denaturation by heating, followed by electrophoretic separation. Radioactive gels were exposed to a phosphor screen for 5 hours at −20 °C and imaged using a Typhoon imaging system (GE). Reactions investigating the fate of the *Sen*DRT9-encoded RT enzyme were analyzed using 10% SDS-PAGE gels. Samples were mixed with 6× SDS Loading Due prior to denaturation by heating and electrophoretic separation. Gels were stained with Coomassie Blue. Uncropped gel and blot images are provided in Supplementary Figure 1.

### Biochemical competition assays with *Sen*DRT9, ExoI, Nuclease P1, and gp58.

Competition assays involving gp58 and ExoI were performed in Polymerization Buffer I. Reactions initially contained 20 nM RT-ncRNA complex and 100 μM dATP, and were first incubated at 37 °C for 10 min, prior to treatment with ExoI in the absence or presence of gp58. ExoI was present at 0.015 Units/μL, and gp58 was titrated into reactions at a range of 0.1–10 μM. Competition assays involving gp58 and Nuclease P1 were performed in Polymerization Buffer II. Reactions initially contained 20 nM RT-ncRNA complex and 100 μM dATP, and were first incubated at 37 °C for 10 min, prior to treatment with Nuclease P1 in the absence or presence of gp58. Nuclease P1 was present at 2 Units/μL, and gp58 was titrated into reactions at a range of 0.1–10 μM. Competition assays using the U_4_>A_4_ mutant ncRNA were performed using 20 nM RT-ncRNA complex and 100 μM [α-^32^P]-dTTP supplemented with cold dTTP. The same procedure was followed as described above, with the exception that the polymerization reaction was carried out for 60 min at 37 °C. Reactions were quenched by proteinase K treatment, prior to addition of 2× RNA Loading Dye (NEB), denaturation by heating, and electrophoretic separation by denaturing 5% urea-PAGE. Uncropped gel and blot images are provided in Supplementary Figure 1.

### Cryo-EM sample preparation.

For all samples, Quantifoil copper grids with a mesh size of 300 and R1.2/1.3 hole spacing were glow discharged using a Pelco EsiGlow at 15 mA for 45 seconds. 3 μL of 6 μM purified GST-DRT9 (trimer) or 2.5 μM DRT9 (hexamer) were applied to prepared grids in a Vitrobot Mk IV (ThermoFischer) set to 100% humidity and 4 °C. Grids were subjected to double-sided blotting with a force of 5 for 6s before plunge-freezing in liquid ethane. Clipped grids were loaded into autogrid boxes and stored in liquid nitrogen before imaging.

### Cryo-EM data collection.

Both datasets were collected on a Talos Arctica (Thermo Fischer) equipped with a Gatan K3 direct electron detector at Montana State University’s Cryo-EM core facility using SerialEM^56^ (v.4.2.0) under the control of SmartScope^57^ (v0.9.4) for automated data collection. 10,450 and 15,594 micrographs were collected for the hexamer and trimer datasets, respectively. All particles were imaged with a pixel size of 0.9061 Å and apparent magnification of 45,000×. The total dose per exposure was calculated as 59.5 (trimer) and 59.8 (hexamer) electrons/sq.Å/exposure.

### Cryo-EM image analysis, *Sen*DRT9 RT-ncRNA trimer.

All image analysis was completed in cryoSPARC^58^ (v4.6.2). After filtering by CTF-fit < 8 Å, and max in-frame motion of 5, 9,814 exposure remained. cryoSPARC Live’s blob picker (50–150 Å particle diameter) was used to identify particles which were then extracted with a box size of 496 px and fourier cropped to 248 px (binx2). 1,878,603 particles were selected from streaming 2D classification and processed to yield a stack of 610,556 particles that were used to generate a 3.7 Å resolution reconstruction without imposing symmetry (Extended Data Fig. 6b). This volume was used to generate *de novo* templates for particle picking which yielded 5,235,086 initial picks. 2,530,970 particles were selected from 2-D classification (Extended Data Fig. 6c) and fed into a 4 class *ab initio* reconstruction (Extended Data Fig. 6d), followed by heterogenous refinement. Non-uniform refinement of the best class produced a 3.7 Å volume containing 2,226,754 particles. After re-extracting unbinned particles and an additional round of 2D classification and 3D sorting by *ab initio* and heterogenous refinement, an initial consensus volume was obtained from 2,107,864 particles. A 5-class 3D classification of these particles without a mask and with force hard classification enabled the removal of additional junk, leaving 1,368,451 particles for further processing. A final round of multi-class *ab initio* reconstruction, followed by heterogeneous refinement (Extended Data Fig. 6e) yielded a final stack of 367,640 particles that were refined to 3.02 Å resolution by C3 non-uniform refinement (EMD-59295) and was used for model building (Extended Data Fig. 6g–i).

### Cryo-EM image analysis, *Sen*DRT9 RT-ncRNA hexamer.

After filtering by CTF-fit < 8 Å resolution, 9,529 exposures remained. 151,584 particles were picked on-the-fly using cryoSPARC Live (100–200 Å particle diameter) and extracted with a box size of 496 px and binned ×4 to expedite processing. A preliminary reconstruction was generated from 48,338 (re-extracted, unbinned) particles that refined to 3.32 Å when D3 symmetry was imposed (3.73 Å, C1). *De novo* templates were produced using this volume, and template picking on the full dataset yielded 6,011,113 picks. After two rounds of 2D classification, 1,441,369 particles (Extended Data Fig. 7c) were re-extracted without binning and a box size of 496 px before removing 206,158 duplicate particles. The remaining 1,227,994 particles were subjected to 7-class *ab initio* reconstruction (Extended Data Fig. 7d) followed by heterogenous refinement. The best class from heterogenous refinement contained 584,077 particles. These particles were re-extracted to obtain better estimates of particle centers before a final round of *ab initio* and heterogenous refinement yielded a stack of 309,844 particles that refined to 2.9 Å resolution by C1 refinement. After volume alignment, imposing D3 symmetry during refinement increased the resolution to 2.59 Å (Extended Data Fig. 7f–h). This map (EMD-59293) was used for model building the hexameric DRT9 ribonucleoprotein complex.

### Cryo-EM model building.

An initial model for the DRT9 trimer was generated using several copies of an AlphaFold3^40^ structure prediction of the RT monomer and fitting them into the final reconstructions using ChimeraX’s^41^ fit in map command. To model the ncRNA, density maps were submitted to ModelAngelo^59^ (v1.0.13), along with the ncRNA sequence identified from RIP-seq experiments described above. While ModelAngelo was unable to build a complete chain for the ncRNA, it correctly modelled a poly-G stretch (80–86) (Extended Data Fig. 8f), which was used as a seed sequence to manually build the rest of the chain in COOT^60^. After building the ncRNA, the complete model for the trimer was refined into the raw map using Phenix (v1.21.2–5419) RealSpaceRefinement^61^ with reference model, secondary structure, and NCS restraints enabled. Iterative editing in COOT (v0.9.8.1) and refinement in Phenix was used to produce the final model (PDB 9NLX). Sharpened maps from Phenix’s anisotropic half-map sharpening tool were used to aid in modelling difficult regions. All outlier residues in the validation report were inspected manually before submission.

The Trimer PDB was used as a starting point for model building of the hexameric form of the complex. After fitting two copies of the trimer structure into the map of the hexamer using ChimeraX’s fit in map command, the model was subjected to RealSpaceRefinement as described above. Iterative editing in COOT and refinement in Phenix were used to produce the final model for the DRT9 hexamer (PDB 9NLV). Sharpened maps from Phenix’s anisotropic half-map sharpening tool were used to aid in modelling difficult regions. All outlier residues were manually inspected before submission. To better visualize unmodelled density near the active site, a 3.0 Å calculated map was generated using ChimeraX’s molmap command and subtracted from the experimental density (Extended Data Fig. 8d).

### RT complex interactome maps.

RT interactome maps were generated by assessing contacts between protomeric and ncRNA subunits with the *contacts* command in ChimeraX^41^, using default settings. Interactions were then plotted with BioCircos^62^.

### Escaper phage isolation.

To isolate escaper phages, serial dilutions of phages T5 or Bas37 were mixed in molten soft agar with *E. coli* K-12 strain MG1655 expressing *Sen*DRT9, *Psa*DRT9, or an empty vector control, and plated on LB-agar plates supplemented with chloramphenicol (25 μg/ml). The plates were incubated overnight at 37 °C to allow plaques to form. Individual plaques were picked into 1 mL of MG1655 culture at OD_600_ ~0.1. Phages forming plaques on *Sen*DRT9 or *Psa*DRT9 plates were inoculated into respective MG1655 cultures expressing *Sen*DRT9 or *Psa*DRT9, while plaques from empty-vector plates were inoculated into MG1655 carrying the empty vector. Cultures were incubated at 37 °C with shaking for 3 hours, and then 800 μL of each culture was transferred to new tubes and mixed with chloroform (5% final volume) to lyse residual bacteria. Cell debris and chloroform were removed by centrifugation for 5 min at 4,000 x *g*, and 5 μL of each phage lysate was propagated further in fresh cultures at OD_600_ ~0.1. Phages were amplified in this manner for a total of 3 rounds. Escaper phage phenotypes were validated via small-drop plaque assays. T5 escaper phages were isolated from *Sen*DRT9 plates and *Psa*DRT9 plates, and Bas37 escaper phages from *Psa*DRT9 plates; attempts to isolate Bas37 escapers from *Sen*DRT9 were unsuccessful.

### Escaper phage whole-genome sequencing.

Phage DNA was isolated by treating 88 μL of phage supernatant with 1 μL of DNase I (200 U/mL, NEB) and 1 μL of RNase A (10 mg/mL, Thermo Fisher Scientific) in 10 μL 1× DNase buffer (NEB). Reactions were incubated at 37 °C for 1 hour, and enzymes were inactivated by heating at 75 °C for 10 min. Phage capsids were digested by adding 1 μL of Proteinase K (20 mg/mL, Thermo Fisher Scientific) and 99 μL of phage lysis buffer (10 mM Tris-HCl, 10 mM EDTA, 0.5% SDS), followed by incubation at 37 °C for 30 min and 55 °C for 30 min. DNA cleanup was performed using Mag-Bind Total Pure NGS beads (Omega), using a bead ratio of 0.9× sample volume.

Phage genomic DNA was tagmented using TnY (a homolog of Tn5) purified in-house following previous methods^63^. 10 ng of purified genomic DNA (gDNA) was tagmented with TnY preloaded with Nextera Read 1 and Read 2 oligos (Supplementary Table 6), followed by proteinase K treatment (NEB, final concentration 16 U/mL) and column purification. PCR amplification and Illumina barcoding was done for 13 cycles with KAPA HiFi Hotstart ReadyMix, with an annealing temperature of 63 °C and an extension time of 1 min. The PCR reactions were then pooled and resolved on a gel. A smear from 400–800 bp was extracted with a Qiaquick Gel Extraction kit (Qiagen) for sequencing on an Element AVITI in paired end mode with 150 cycles per end.

Resulting datasets were processed using cutadapt^42^ (v4.2) to remove Illumina adapter sequences, trim low-quality ends from reads, and filter out reads shorter than 15 bp. Reads were mapped to the T5 (NC_005859.1) genome or Bas37 genome (MZ501089.1), using Bowtie2^64^ (v2.2.1) with default parameters. SAMtools^44^ (v1.17) was used to sort and index alignments, and coverage tracks were visualized in IGV^46^ (v2.17.4). Mutations were identified with breseq^65^ (v.0.39.0) using alignments from Bowtie2 and the same T5 or Bas37 reference genomes as above. T5 and Bas37 escaper mutations are listed in Supplementary Table 3.

### Infection response growth curves.

*E. coli* K-12 strain MG1655 cells transformed with plasmids encoding WT or MUT *Sen*DRT9 were grown to OD_600_ of 0.2 in LB media supplemented with chloramphenicol (25 μg/mL). 180 μL of each culture was transferred into wells of a 96-well optical plate containing 20 μL of T5 lysate diluted to result in a final MOI of 5 or 0.05, or 20 μL of LB for the uninfected condition. The plate was incubated for 4 hours at 37°C with shaking. OD_600_ values were recorded every 10 min using a Synergy Neo2 microplate reader (Biotek).

### Cell viability assays with trigger and host factor candidates.

To assess the effects of *Sen*DRT9 co-expression with phage genes of interest on cell growth, candidate trigger genes identified by escaper screening were cloned onto an expression vector under the control of an arabinose-inducible araBAD promoter. *E. coli* K-12 strain MG1655 cells expressing either WT or MUT *Sen*DRT9 were transformed with trigger plasmids and grown under repressive conditions in LB media supplemented with 2% glucose. Overnight cultures were centrifuged at 4,000 x *g* for 5 min and cell pellets were washed with fresh LB to remove residual glucose. Cells were pelleted again as before and resuspended in fresh LB. 10× serial dilutions of each culture were prepared and 5 μL were spotted on LB agar plates supplemented with chloramphenicol (25 μg/mL), spectinomycin (100 μg/mL), and arabinose (0.2%). Plates were incubated overnight at 37 °C and colonies were counted the next day. CFU/mL were calculated as described above for the colony formation time course.

To assess cell viability of Δ*sbcB* MG1655 cells expressing *Sen*DRT9, a Δ*sbcB* knock-out strain were transformed with either WT *Sen*DRT9 or MUT *Sen*DRT9. Cells were recovered in 10 mL of LB media at 37 °C for 4 hours. Following recovery, cultures were diluted 1:10 in fresh LB, and 100 μL were plated on LB agar plates supplemented with chloramphenicol and kanamycin. CFU/mL were calculated using the following formula: $\frac{number\;of\;colonies}{0.1\;mL\;x\;dilution\;factor}$.

### Nucleotide quantification by LC-MS/MS.

*E. coli* K-12 strain MG1655 cells were transformed with empty vector (EV) or *Sen*DRT9 plasmids. Individual colonies were inoculated into LB media supplemented with chloramphenicol (25 μg/mL) and grown overnight at 37 °C with shaking. The following day, cultures were diluted 1:100 in 20 mL fresh LB media and grown to OD_600_ of 0.3. Cultures were split in half and T5 phage was added to one half at an MOI of 2. Uninfected and infected cultures were grown for an additional 45 minutes at 37 °C with shaking. Cells were centrifuged at 4,000 x *g* for 5 min at 4 °C and pellets were washed with 1 mL of cold 1× TBS. Cells were centrifuged again at 15,000 x *g* for 1 min at 4 °C. After supernatants were removed, pellets were flash-frozen in liquid nitrogen and stored at −80 °C.

Cell lysates were prepared as previously described^8^. Flash-frozen pellets were thawed on ice and resuspended in 600 μL of 100 mM sodium phosphate buffer (10:1 mixture of Na_2_HPO_4_ and NaH_2_PO_4_) supplemented with 1 mg mL^−1^ lysozyme. Cells were mechanically lysed using a bead beater (2.5 min × 2 cycles at 4 °C). Lysates were centrifuged at 4,000 x *g* for 15 min at 4 °C and supernatants were transferred to new tubes. Samples were transferred to Amicon Ultra-0.5 3 kDa filter units (Merck Millipore) and centrifuged at 12,000 x *g* for 45 min at 4 °C. Filtrates containing metabolites were collected and stored at −80 °C. Upon retrieval from −80 °C, and prior to use for injection in LC-MS/MS, filtrates were additionally purified by centrifugation at 20,000 x *g* for 20 min at 4 °C. To confirm that each condition contained equal amounts of cellular material, the protein concentration in filtration supernatants was measured by BCA assay (Thermo Fisher Scientific). Protein concentrations varied by < 10% across all samples.

Nucleotide and deoxynucleotide profiling analysis was carried out with ion-paired reversed phase liquid chromatography using an HPLC (1290 Infinity II, Agilent Technologies) coupled to a triple quadrupole mass spectrometer (6495D, Agilent Technologies) with electrospray ionization operated in negative mode. The column was a ZORBAX RRHD Extend-C18 (2.1 × 150 mm, 1.8 μm pore size; 759700–902, Agilent Technologies). 5 μL of the experimental samples were injected. Mobile phase A was 3% methanol (in H_2_O), and mobile phase B was 100% methanol. Both mobile phases contained 10 mM of the ion-pairing agent tributylamine (90780, Sigma Aldrich), 15 mM acetic acid, and 5 μM medronic acid (5191–4506, Agilent Technologies). The LC gradient was: 0–2.5 min 100% A, 2.5–7.5 min ramp to 80% A, 7.5–13 min ramp to 55% A, 13–20 min ramp to 99% B, 20–24 min hold at 99% B. Flow rate was 0.25 mL/min, and the column compartment was heated to 35°C. The column was then backflushed with 100% acetonitrile (0.25 mL/min flow rate 24.05–27 min, followed by 0.8 mL/min flow rate 27.5–31.35 min, and 0.6 mL/min flow rate 31.35–31.50 min) and re-equilibrated with 100% A (0.4 mL/min flow rate 32.25–40 min). The conditions of the MRM transitions for dATP and dTTP were as follows (collision energy (V)): dATP, 490>391.9 (24), 490>158.9 (32); dTTP, 481>383.1 (20), 481>158.8 (36). Fragmentor and CAV were kept constant at 166 V and 4 V, respectively. For other nucleotides and deoxynucleotides measured, conditions of the MRM transitions are shown in Supplementary Table 7. In addition, the identity of each compound was confirmed with subsequent injection and acquisition of a pure chemical standard. Data analysis, including peak area integration and signal extraction, was performed with Skyline-daily (v24.1.1.398).

### Nucleoside supplementation assays in plaque assays.

Nucleoside supplementation was performed using *E. coli* MG1655 cells transformed with either empty vector or DRT9 expression plasmids. Cultures were grown in LB supplemented with chloramphenicol (25 μg/mL) until OD_600_ reached 0.3. Deoxythymidine, deoxyadenosine, deoxycytidine or deoxyguanosine were added to 4 mL cultures at a final concentration of 20 μM. Cultures were pre-incubated at 37 °C with shaking for 5 min, before adding phage T5 at MOI of 2.5, and grown for another hour. Phages were isolated by adding chloroform to a final concentration of 4%, and samples were centrifuged at 3,000 x *g* for 3 min. Supernatant was used to make 10× serial dilutions used for small-drop plaque assays, performed as described previously. EOP was calculated by spotting the phage on soft agar containing *E. coli* MG1655 cells transformed with an empty vector; the graph shows results from two technical replicates.

### Co-immunoprecipitation Western blot.

*E. coli* K-12 strain MG1655 cells were transformed with plasmids encoding either untagged *Sen*DRT9, *Sen*DRT9 with mutated RT catalytic residues (YAAA), N-term 3xFLAG-tagged *Sen*DRT9, or N-term 3xFLAG-tagged *Sen*DRT9 with mutated RT catalytic residues (YAAA), as well as a plasmid encoding C-term V5-tagged gp58. Individual colonies were inoculated into LB media supplemented with chloramphenicol (25 μg/mL), spectinomycin (100 μg/mL) and 2% glucose and grown overnight at 37 °C with shaking. The following day, cultures were diluted 1:100 in 25 mL fresh LB media with continued 2% glucose repression and grown to OD600 of 0.4. Cultures were centrifuged at 4,000 x *g* for 5 min and cell pellets were washed with fresh LB to remove residual glucose. Cells were pelleted again as before, and resuspended in fresh LB with chloramphenicol (25 μg/mL), spectinomycin (100 μg/mL) and 0.2% arabinose, and grown at 37 °C with shaking for 30 min. Cells were centrifuged at 4,000 x *g* for 10 min at 4 °C and pellets were washed with 1 mL of cold 1× TBS. After another round of centrifugation at 15,000 x *g* for 1 min at 4 °C supernatants were removed, and pellets were flash-frozen in liquid nitrogen and stored at −80 °C.

To prepare antibody–bead complexes for immunoprecipitation, Dynabeads Protein G (Thermo Fisher Scientific) were washed 3× in 1 mL IP-MS lysis buffer (50 mM Tris-HCl pH 7.5 at 25 °C, 150 mM NaCl, 5% glycerol, 0.2% Triton X-100). Washed beads were resuspended again in 1 mL IP-MS lysis buffer, combined with anti-FLAG antibody (Sigma-Aldrich, F3165), and rotated for > 3 hours at 4 °C. 75 μL of beads and 10 μL of antibody were prepared per sample. Antibody-bead complexes were washed 2× to remove unbound antibodies and resuspended in IP-MS lysis buffer to a final volume of 75 μL per sample.

Flash-frozen pellets were thawed on ice and resuspended in 1.2 mL IP-MS lysis buffer supplemented with 1× Complete Protease Inhibitor Cocktail (Roche) and 0.1 U/μL SUPERase•In RNase Inhibitor (Thermo Fisher Scientific). Cells were lysed by sonication (2 sec ON, 5 sec OFF, amplitude 20%, 1.5 min total time) and lysates were cleared by centrifugation at 21,000 x *g* for 15 min at 4 °C. Supernatants were transferred to new tubes and protein concentrations were measured by BCA assay (Thermo Fisher Scientific). For each sample, 1 mg of protein was combined with 75 μL of antibody-bead complex and rotated overnight at 4 °C. 50 μL of each cleared lysate was set aside as input control and stored at −80 °C. The next day, each sample was washed 2× with 1 mL cold IP-MS wash buffer 1 (50 mM Tris-HCl pH 7.5 at 25 °C, 150 mM NaCl, 5% glycerol, 0.02% Triton X-100) followed by another 2 washes with cold IP-MS wash buffer 2 (50 mM Tris-HCl pH 7.5 at 25 °C, 150 mM NaCl, 5% glycerol).

For elution, beads were resuspended in 75 μL 2× SDS loading buffer and incubated at 50 °C for 10 min. Input samples were thawed, and both input and IP samples were prepared for SDS-PAGE by combining 15 μL protein with 18.75 μL 2× SDS loading buffer and 3.75 μL 0.1 M DTT. Samples were denatured at 95 °C for 5 min and immediately transferred to ice. Samples were loaded onto 12% Criterion XT Bis-Tris Protein Gels and subjected to electrophoresis in XT MES running buffer at 80 V for 5 min followed by 200 V for 30–40 min until the dye front reached the bottom of the gel. Proteins were transferred to nitrocellulose membranes using iBlot. Membranes were blocked in TBS-T (20 mM Tris-HCl pH 7.5, 150 mM NaCl, 0.1% Tween) containing 5% BSA for 1 hour at room temperature. Membranes were incubated overnight at 4 °C with V5 primary antibody (V5-Tag (D3H8Q), Cell Signaling Technologies) diluted 1:2000 in the blocking buffer. Following three 5-min washes with TBS-T, membranes were incubated with the secondary antibody (mouse anti-rabbit IgG (L27A9)) diluted 1:5000 in a 1:1 mixture of blocking buffer and TBS-T for 1 hour at room temperature. After three 5-min washes with TBS-T, signals were detected using the West Dura Signal kit (Thermo Fisher Scientific) and imaged using the chemiluminescence setting on an Amersham Imager 600 (GE Healthcare). Uncropped gel and blot images are provided in Supplementary Figure 1.

### Co-immunoprecipitation mass spectrometry.

*E. coli* K-12 strain MG1655 cells were transformed with plasmids encoding either untagged *Sen*DRT9, N-term 3xFLAG-tagged *Sen*DRT9, or N-term 3xFLAG-tagged *Sen*DRT9 with mutated RT catalytic residues (YAAA). Individual colonies were inoculated into LB media supplemented with chloramphenicol (25 μg/mL) and grown overnight at 37 °C with shaking. The following day, cultures were diluted 1:100 in 40 mL fresh LB media and grown to OD_600_ of 0.3. Cultures were split in half and T5 phage was added to one half at an MOI of 5. Uninfected and infected cultures were grown for an additional 45 minutes at 37 °C. Cells were centrifuged at 4,000 x *g* for 10 min at 4 °C and pellets were washed with 1 mL of cold TBS. After centrifuging again at 15,000 x *g* for 1 min at 4 °C and removing supernatants, pellets were flash-frozen in liquid nitrogen and stored at −80 °C.

To prepare antibody–bead complexes for immunoprecipitation, Dynabeads Protein G (Thermo Fisher Scientific) were washed 3× in 1 mL IP-MS lysis buffer (50 mM Tris-HCl pH 7.5 at 25 °C, 150 mM NaCl, 5% glycerol, 0.2% Triton X-100). Washed beads were resuspended again in 1 mL IP-MS lysis buffer, combined with anti-FLAG antibody (Sigma-Aldrich, F3165), and rotated for > 3 hours at 4 °C. 100 μL of beads and 10 μL of antibody were prepared per sample. Antibody-bead complexes were washed 2× to remove unbound antibodies and resuspended in IP-MS lysis buffer to a final volume of 100 μL per sample.

Flash-frozen pellets were thawed on ice and resuspended in 1.2 mL IP-MS lysis buffer supplemented with 1× Complete Protease Inhibitor Cocktail (Roche) and 0.1 U/μL SUPERase•In RNase Inhibitor (Thermo Fisher Scientific). Cells were lysed by sonication and lysates were cleared by centrifugation at 21,000 x *g* for 15 min at 4 °C. Supernatants were transferred to new tubes and protein concentrations were measured by BCA assay (Thermo Fisher Scientific). For each sample, 1 mg of protein was combined with 100 μL of antibody-bead complex and rotated overnight at 4 °C. The next day, each sample was washed 2× with 1 mL cold IP-MS wash buffer 1 (50 mM Tris-HCl pH 7.5 at 25 °C, 150 mM NaCl, 5% glycerol, 0.02% Triton X-100) followed by another 2 washes with cold IP-MS wash buffer 2 (50 mM Tris-HCl pH 7.5 at 25 °C, 150 mM NaCl, 5% glycerol). For elution and on-bead tryptic digestion, beads were resuspended in 80 μL Tris-urea buffer (50 mM Tris-HCl pH 7.5 at 25 °C, 2 M urea, 1 mM DTT) with 0.5 μg/mL sequencing grade modified trypsin (Promega) and incubated 1 hr at 25 °C with regular agitation. Supernatants were transferred to new tubes and beads were resuspended again in 60 μL Tris-urea buffer without trypsin. Supernatants were combined with those from the first elution step. This step was repeated once more, resulting in a total eluate volume of 200 μL per sample. Eluates were centrifuged at 5,000 x *g* for 1 min and supernatants were transferred to new tubes to remove any residual beads. Eluates were flash-frozen in liquid nitrogen and stored at −80 °C prior to further processing.

IP-MS eluates were reduced with 5 mM DTT at 25 °C with 600 rpm agitation for 45 minutes, after which they were alkylated in the dark with 10 mM iodoacetamide (IAA) at 25 °C and 600 rpm for 45 minutes. Samples were then cleaned up using the previously described SP3 protocol^66^. Specifically, 500 μg of an equal mixture of carboxylate-modified hydrophilic beads (Cytiva 45152105050250) and hydrophobic beads (Cytiva 65152105050250) was added to each sample. To induce the binding of proteins to the SP3 beads, 100% ethanol was added to the sample at a 1:1 ratio, and the samples were incubated at 25 °C and 1000 rpm for 15 minutes. After incubation, the beads were washed three times with 80% ethanol and reconstituted in freshly prepared 100 mM ammonium bicarbonate. Samples were digested off the beads using 1 μg of sequencing grade modified trypsin (Promega V5111). After 16 hours of incubation at 25 °C and 600 rpm, samples were taken off the magnetic beads, dried down using a Thermo Savant SpeedVac, and reconstituted in 3% acetonitrile/0.2% formic acid.

Digested peptides from the IP-MS eluates were analyzed on a Waters M-Class UPLC using a 15 cm x 75 μm IonOpticks C18 1.7 μm column coupled to a benchtop Thermo Fisher Scientific Orbitrap Q Exactive HF mass spectrometer. Peptides were separated at a 400 nL min^−1^ flow rate with a 90-minute gradient, including sample loading and column equilibration times. Data were acquired in data-dependent mode using Xcalibur (v4.5.474.0.); each cycle’s 12 most intense peaks were selected for MS2 analysis. MS1 spectra were measured with a resolution of 120,000, an AGC target of 3e6, and a scan range from 300 to 1800 m/z. MS2 spectra were measured with a resolution of 15,000, an AGC target of 1e5, a scan range from 200–2000 m/z, and an isolation window width of 1.6 m/z.

Raw data were searched against a combined reference proteome that included the *E. coli* K-12 strain MG1655 proteome (NCBI RefSeq assembly GCF_000005845.2), T5 phage proteome (NCBI RefSeq assembly GCF_000858785.1), and the *Sen*DRT9 RT sequence, using MaxQuant^67^ (v2.0.3.0). One or more unique/razor peptides were required for protein identification. All subsequent analyses were performed in R (v4.4.0).

For peptide-level analysis to compare peptide detection between the immunoprecipitated WT and MUT RT samples, the data were first filtered to remove peptides with LFQ intensity of 0. Values were then normalized to the sum of LFQ intensities for each condition. After log_2_ transformation, significantly depleted peptides were determined by unpaired two-tailed t-test, followed by multiple comparisons correction using the Benjamini-Hochberg method.

For protein-level analysis to identify DRT9 interactors, protein groups with more than five MS/MS spectral counts in at least one condition were retained in the normalized LFQ intensities. A random value between 1 × 10^6^ and 1% of the lowest data point was added as a “pseudocount” to all data points, followed by log_2_ transformation. The data were then used to determine significantly enriched proteins by unpaired two-tailed t-test, with correction for multiple comparisons by the Benjamini-Hochberg method. Significantly enriched interactors were identified using cutoffs of fold-enrichment > 20 and adjusted p-value < 0.05, relative to a control IP from cells expressing untagged WT *Sen*DRT9. IP-MS hits are described in Supplementary Table 8.

### Phylogenetic analysis of DRT9 RT and other reverse transcriptases.

Sequences of DRT9, a Group II intron RT, KpnDRT2, AbiA, AbiK, AbiP2, and the HIV-1 RT were used to predict monomeric and apo structures of each protein with AlphaFold 3^40^. These predicted structures were then aligned with FoldMason^68^, and the regions corresponding to the Palm and Finger domains were extracted from the alignment. FastTree^36^ was then used to build the phylogenetic tree shown in Supplementary Figure 2.

## Extended Data

**Extended Data Table 1:** Cryo-EM data collection, refinement, and validation statistics

	#1 GST-DRT9 Trimer (EMDB-49525) (PDB 9NLX)	#2 DRT9 Hexamer, Pre-Polymerization State (EMDB-49523) (PDB 9NLV)

**Data collection and processing**		
Magnification	45,000	45,000
Voltage (kV)	200	200
Electron exposure (e−/Å^2^)	59.5	59.8
Defocus range (μm)	−0.8, −2.5	−0.8, −2.5
Pixel size (Å)	.9061	.9061
Symmetry imposed	C3	D3
Initial particle images (no.)	5,235,086	6,029,711
Final particle images (no.)	367,640	309,844
Map resolution (Å)	3.02	2.59
FSC threshold	0.143	0.143
Map resolution range (Å)	2.6–6.0Å	2.0–4.5
**Refinement**		
Initial model used (PDB code)	AlphaFold3, ModelAngelo	AlphaFold3, ModelAngelo
Map sharpening methods	Phenix Half-Map	Phenix Half-Map
Refinement Package	Phenix	Phenix
Refinement Method	RealSpaceRefinement	RealSpaceRefinement
**Model composition**		
Non-hydrogen atoms	20,076	41,424
Protein residues	1,443	2,952
Nucleotide Residues	441	882
Ligands	0	0
***B* factors (Å^2^)**		
Protein	54.73	86.41
Nucleotide	75.05	145.79
Ligand	N/A	N/A
**R.m.s. deviations**		
Bond lengths (Å)	0.003 (0)	0.004 (0)
Bond angles (°)	0.772 (0)	0.694 (0)
**Validation**		
MolProbity score	1.67	1.33
Clashscore	8	9
Poor rotamers (%)	0.5(5)	0(1)
**Ramachandran plot**		
Favored (%)	99	98
Allowed (%)	1	2
Disallowed (%)	0	0

This table lists statistics related to cryo-EM structures of the *Sen*DRT9 RT-ncRNA complex.

## Supplementary Material

DRT9_SuppFig1-2

DRT9_SuppInfoGuide

DRT9_SuppTables1-8

## Data Availability

Next-generation sequencing data are available in the National Center for Biotechnology Information Gene Expression Omnibus (NCBI GEO) under the accessions GSE295895, GSE295896, and GSE295897. The published genomes used for bioinformatics analyses were obtained from NCBI (Supplementary Table 1). Cryo-EM maps and experimentally determined models were deposited to the appropriate public database (i.e. EMDB, PDB) under the accessions EMD-49525, PDB:9NLX (GST-DRT9 Trimer) and EMD-49523, PDB:9NLV (DRT9 Hexamer). Previously determined protein structures used for structural comparisons were obtained from the PDB (7R06; 8FAK). The phylogenetic tree of UG RT homologs was obtained from https://doi.org/10.1093/nar/gkac467. Mass spectrometry data are available in the MassIVE database under the accession MSV000097825. Source data is available for Figures 1e, 1g, 1i, 2b, 2c, 2d, 5c, 5d, 5h, 6a, 6e, and Extended Data Figures 1b, 1e, 1f, 2f, 2g, 3d, 3f, 4b, 4e, 5b, 5c, 9b, 9c. All other data are available in the manuscript or in Supplementary Information.
