# Supplementary material for: Protein-primed homopolymer synthesis by an antiviral reverse transcriptase
Source: Nature. Author manuscript; Available in PMC 2025 Sep 30. (PMC12483538; doi:10.1038/s41586-025-09179-5)

Supplementary Figure 1 | Uncropped images of electrophoretic separation assays.

Dashed boxes outline the cropped area used in the indicated figures.

Fig. 1h (left)

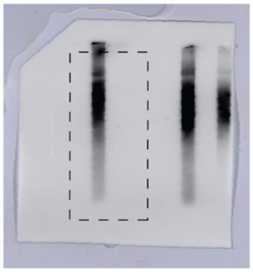

Fig. 1h (right)

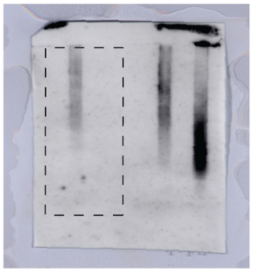

Fig. 2f (left)

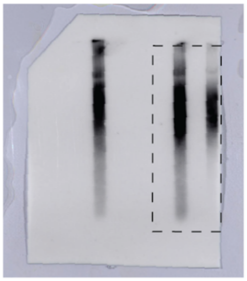

Fig. 2f (right)

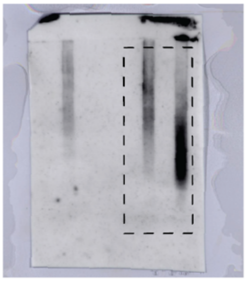

Fig. 3a

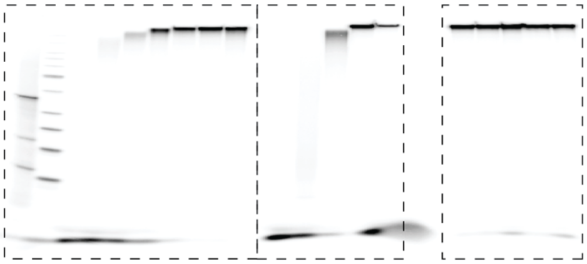

Fig. 3b

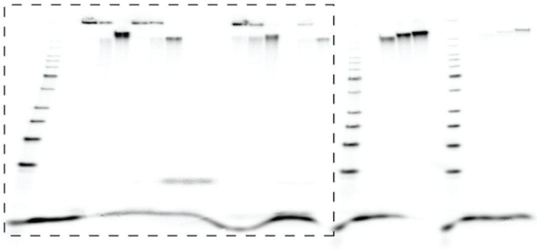

Fig. 3c (left)

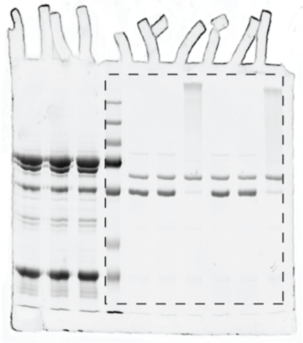

Fig. 3c (right)

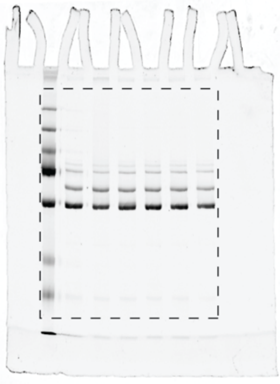

Fig. 3f

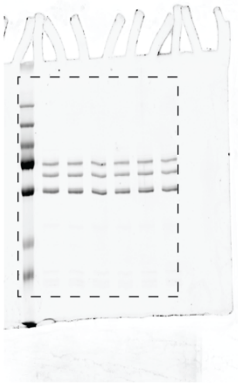

Fig. 3g

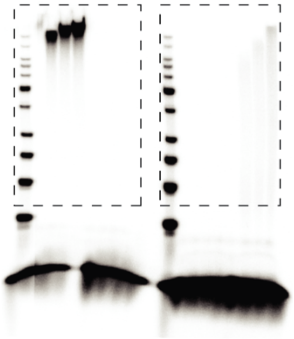

Fig. 5f

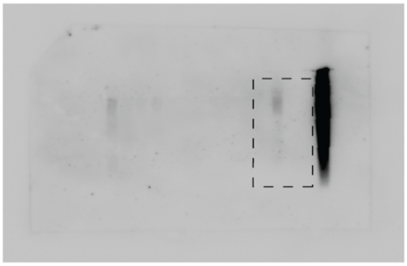

Fig. 6b

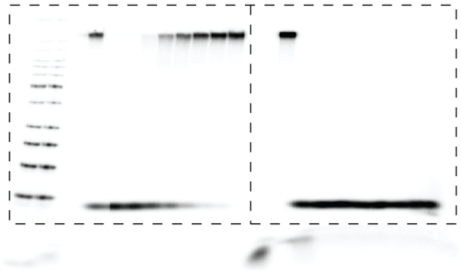

Extended Data Fig. 2d

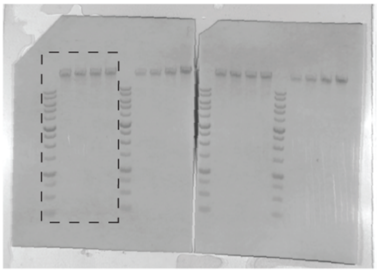

Extended Data Fig. 3g

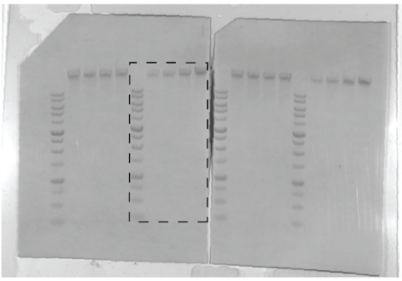

Extended Data Fig. 4b

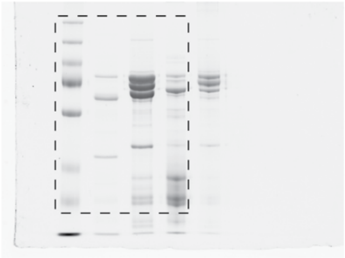

Extended Data Fig. 4c

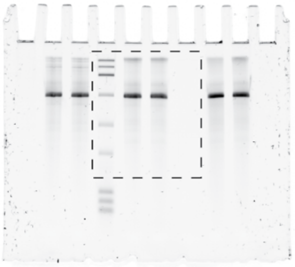

Extended Data Fig. 4f

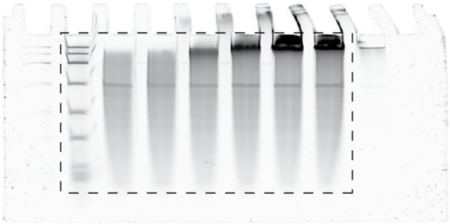

Extended Data Fig. 5a (left)

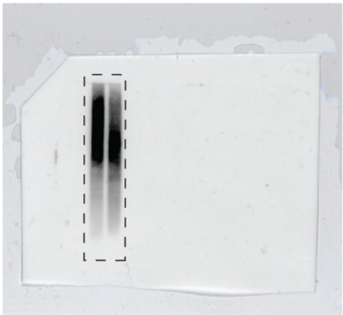

Extended Data Fig. 5a (right)

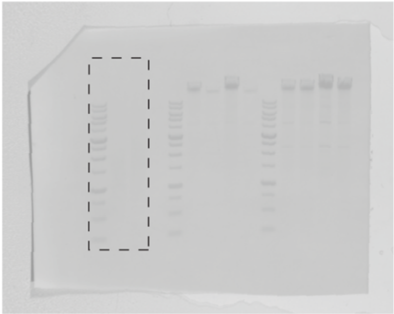

Extended Data Fig. 5f

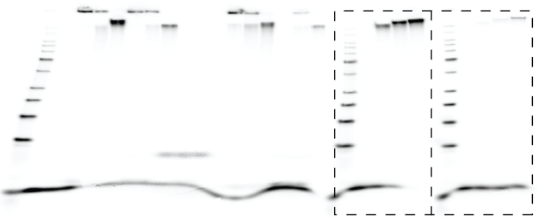

Extended Data Fig. 5g (left)

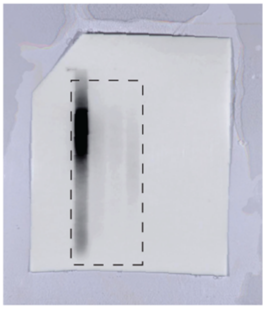

Extended Data Fig. 5g (right)

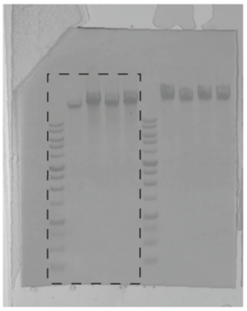

Extended Data Fig. 5h

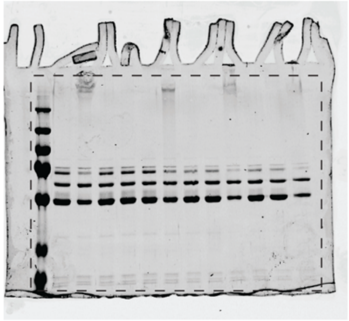

Extended Data Fig. 5i

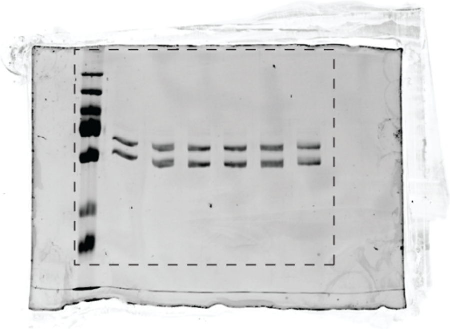

Extended Data Fig. 5j (left)

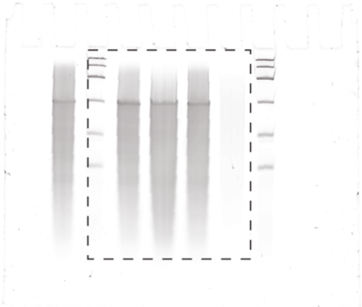

Extended Data Fig. 5j (right)

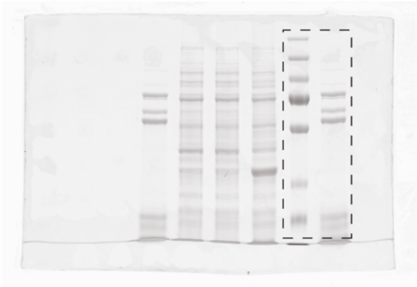

Extended Data Fig. 6j

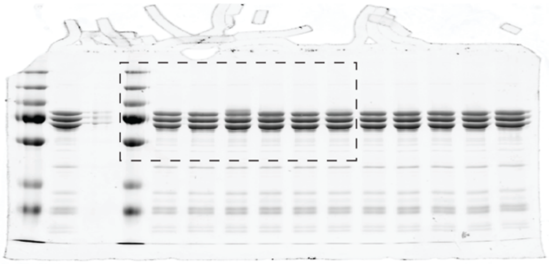

Extended Data Fig. 9d

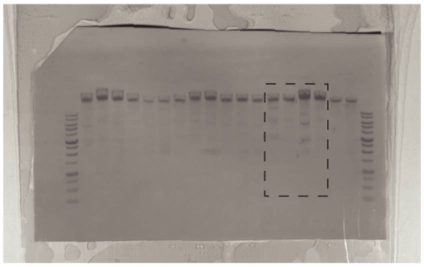

Extended Data Fig. 9e

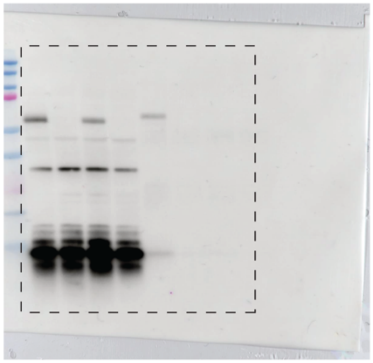

Extended Data Fig. 9f

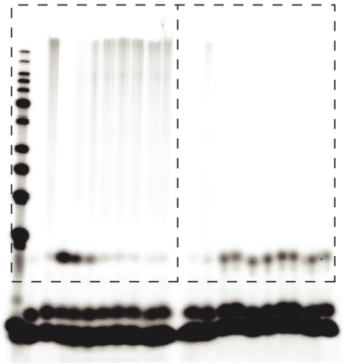

Supplementary Figure 2 | Comparison of domain composition and 3D structure across evolutionarily diverse RT homologs.

a, Phylogenetic tree of palm-finger domains of the indicated RT enzymes (left), shown alongside their domain composition (middle) and monomeric structure (right). The tree is rooted to the HIV RT as an outgroup; PDB IDs are shown, alongside the new structure of the *Sen*DRT9 RT presented in this study (top right). **b,** Structural comparison of the indicated bacterial RT multimeric complexes (left), with the hexameric *Sen*DRT9 RT-ncRNA complex shown at the top. AbiA, AbiK, and AbiP2 complexes rely exclusively on protein-protein interactions, whereas the ncRNA plays a crucial role in DRT9 hexamer assembly, as highlighted in the interactome plots (right). Protein domains and ncRNA are colored according to the legend shown at the bottom.

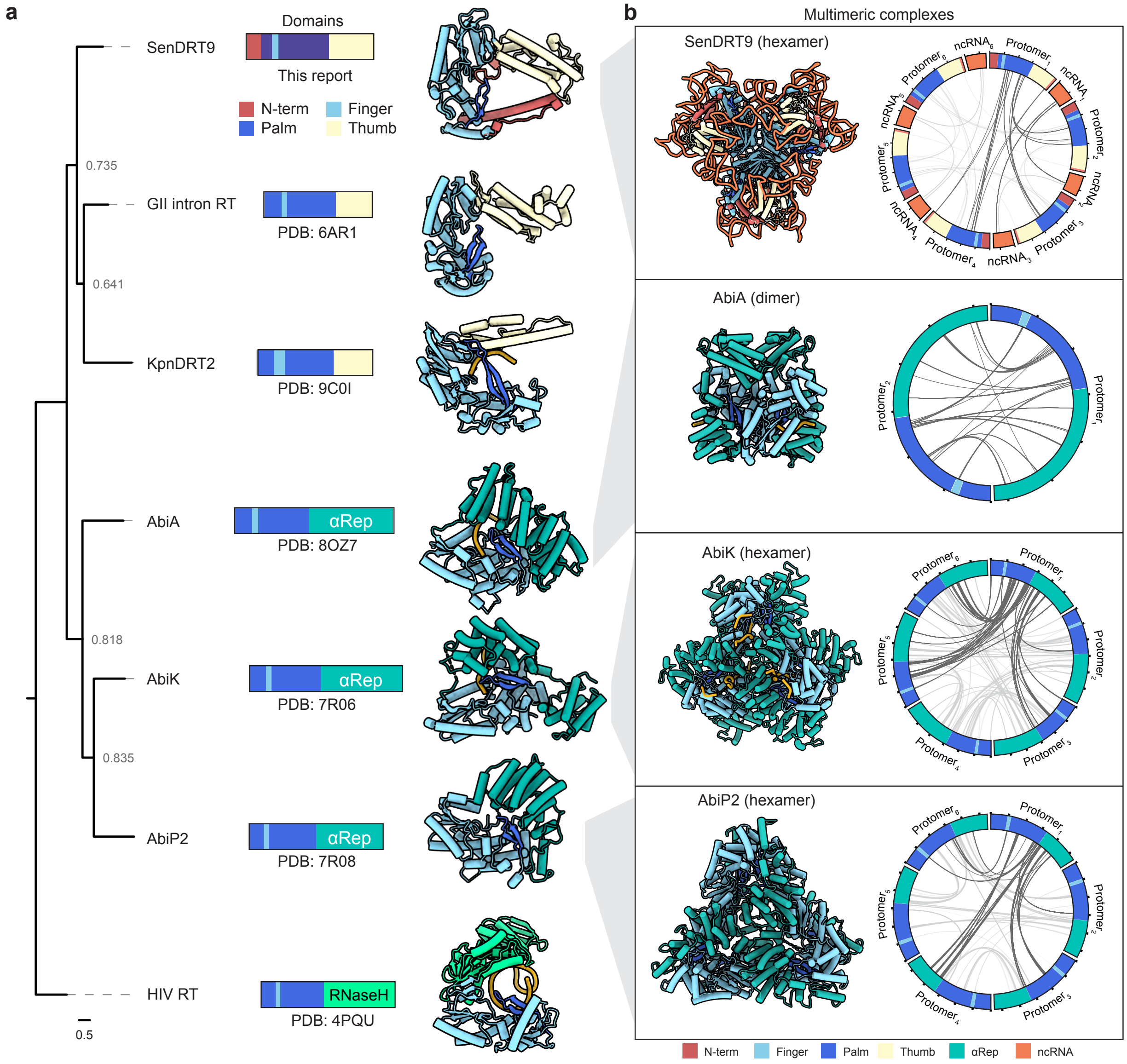

Supplement: DRT9_SuppFig1-2 [file NIHMS2110959-supplement-DRT9_SuppFig1-2.pdf]
