# Supplementary material for: Protein-primed homopolymer synthesis by an antiviral reverse transcriptase
Source: Nature. Author manuscript; Available in PMC 2025 Sep 30. (PMC12483538; doi:10.1038/s41586-025-09179-5)
Supplement: DRT9_SuppInfoGuide [file NIHMS2110959-supplement-DRT9_SuppInfoGuide.pdf]

Supplementary Information

Protein-primed homopolymer synthesis by an antiviral reverse transcriptase

Stephen Tang[1]*, Rimantė Žedaveinytė[1]*, Nathaniel Burman[2]*, Shishir Pandey[2]*, Josephine L. Ramirez[1], Louie M. Kulber[1], Tanner Wiegand[1,3], Royce A. Wilkinson[2], Yanzhe Ma[4], Dennis J. Zhang[1†], George D. Lampe[1,3], Mirela Berisa[5], Marko Jovanovic[4], Blake Wiedenheft[2‡], Samuel H. Sternberg[1,3‡]

[1]Department of Biochemistry and Molecular Biophysics, Columbia University, New York, NY, USA.

[2]Department of Microbiology and Cell Biology, Montana State University, Bozeman, MT, USA.

[3]Howard Hughes Medical Institute, Columbia University, New York, NY, USA.

[4]Department of Biological Sciences, Columbia University, New York, NY, USA.

[5]Metabolomics Core, Icahn School of Medicine at Mount Sinai, New York, NY, USA.

*These authors contributed equally to this work.

[†]Present address: Section of Microbiology, Department of Biology, University of Copenhagen, Copenhagen, Denmark.

[‡]Corresponding authors. Email: bwiedenheft@gmail.com; shsternberg@gmail.com

Supplementary Figures

Supplementary Figure 1 | Uncropped images of electrophoretic separation assays and LB-agar culture plates.

Supplementary Figure 2 | Comparison of domain composition and 3D structure across evolutionarily diverse RT homologs.

Supplementary Tables

Supplementary Table 1 | DRT9-encoded RT homologs in Extended Data Fig. 1a phylogenetic tree.

Supplementary Table 2 | List of DRT9-family immune systems tested in this study.

Supplementary Table 3 | Genotypes of escaper phages that evade DRT9 immunity.

Supplementary Table 4 | Strains used in this study.

Supplementary Table 5 | Description and sequence of plasmids used in this study.

Supplementary Table 6 | Probes and oligonucleotides used in this study.

Supplementary Table 7 | Conditions of MRM transitions from metabolomics measurements.

Supplementary Table 8 | IP-MS hits plotted in Fig. 6d.
